# Invasive Trichosporon Infection: a Systematic Review on a Re-emerging Fungal Pathogen

**DOI:** 10.3389/fmicb.2016.01629

**Published:** 2016-10-17

**Authors:** João N. de Almeida Júnior, Christophe Hennequin

**Affiliations:** ^1^Central Laboratory Division-LIM03, Faculdade de Medicina da Universidade de São PauloSão Paulo, Brazil; ^2^Laboratory of Medical Mycology-LIM53, Instituto de Medicina Tropical da Universidade de São PauloSão Paulo, Brazil; ^3^Service de Parasitologie-Mycologie-AP-HP, Hôpital St AntoineParis, France; ^4^Institut National de la Santé et de la Recherche Médicale UMR 1135, Centre National de la Recherche Scientifique ERL 8255, Sorbonne Universités, University Pierre and Marie Curie (UPMC)Paris, France; ^5^Centre d'Immunologie et des Maladies Infectieuses, Bd de l'hôpitalParis, France

**Keywords:** *Trichosporon* species, invasive infection, risk factors, prognosis, diagnosis, treatment, antifungal resistance

## Abstract

**Objectives:** This review aimed to better depict the clinical features and address the issue of therapeutic management of *Trichosporon* deep-seated infections.

**Methods:** We comprehensively reviewed the cases of invasive *Trichosporon* infection reported in the literature from 1994 (date of taxonomic modification) to 2015. Data from antifungal susceptibility testing (AST) studies were also analyzed.

**Results:** Two hundred and three cases were retained and split into four groups: homeopathy (*n* = 79), other immunodeficiency conditions (*n* = 41), miscellaneous (*n* = 58) and newborns (*n* = 25). *Trichosporon asahii* was the main causative species (46.7%) and may exhibit cross-resistance to different antifungal classes. The unfavorable outcome rate was at 44.3%. By multivariate analysis, breakthrough infection (OR 2.45) was associated with unfavorable outcome, whilst the use of an azole-based therapy improved the prognosis (OR 0.16). Voriconazole-based treatment was associated with favorable outcome in hematological patients (73.6 vs. 41.8%; *p* = 0.016). Compiled data from AST demonstrated that (i) *T. asahii* exhibits the highest MICs to amphotericin B and (ii) voriconazole has the best *in vitro* efficacy against clinical isolates of *Trichosporon* spp.

**Conclusions:** *Trichosporon* infection is not only restricted to hematological patients. Analysis of compiled data from AST and clinical outcome support the use of voriconazole as first line therapy.

## Introduction

*Trichosporon* spp. are yeast-like organisms responsible for superficial infection (white piedra), allergic pneumonitis and more rarely, invasive infection (Colombo et al., 2011). In the 1980's, *Trichosporon* invasive infections (ITI) were considered the second most common cause of fungemia in patients with hematological malignancies (Walsh et al., 1986). However, as triazole derivatives, such as fluconazole, became available, the incidence of those infections decreased (Kaufman et al., 2001; Gomes et al., 2014). On the contrary, maybe due to the breakthrough of the echinocandins, now considered as drugs of choice in many clinical contexts at high-risk for invasive fungal infection (IFI), we now deal with a reemergence of this difficult to manage pathogen (Liao et al., 2015). Indeed, the diagnosis of these infections is difficult to anticipate, largely due to the lack of a specific biomarker assay. In addition, identification at the species level is sometimes confusing since the in-depth taxonomic revision of the genus proposed in 1994 (Guého et al., 1994). From then, molecular analyses led to the definition of no less than 50 accepted species within the genus, 17 of which being medically relevant (Hickey et al., 2009; Colombo et al., 2011). Therapeutic management of those infections may also be challenging, since *Trichosporon* spp. exhibit an intrinsic resistance to the echinocandins and a poor susceptibility to the polyenes (Walsh et al., 1990).

To improve our knowledge of the epidemiology, diagnosis and therapeutic management of those infections, we performed a comprehensive review of the case reports and series published between 1994 and 2015. In addition, we also analyzed studies of *in vitro* susceptibility testing including a significant number of *Trichosporon* isolates.

## Materials and methods

### Search strategy and selection criteria

We performed by the end of 2015 a systematic search in the Pubmed database using the following key words: “*Trichosporon*,” “trichosporonosis,” “invasive infection,” “sepsis,” “deep-seated infection” and “fungemia.” To potentially detect species-specific epidemiological traits, we limited our search after 1994, date of the revised taxonomic classification. We only considered proven invasive infections based on the EORTC-MSG definitions (De Pauw et al., 2008). The cases of probable *Trichosporon* pneumonia were also considered using the criteria proposed by Colombo et al. (2011). Fungemia and infections involving two non-contiguous organs were considered as disseminated. Data on age, sex, underlying disease, immunological status, and the use of intravenous catheter were collected for each case. Neutropenia was defined as an absolute neutrophil count ≤ 0.5 × 10^9^ neutrophils/L at the time of *Trichosporon* isolation. Antibiotic therapy was taken into account as a possible predisposing factor when administered for at least 1 week prior to the isolation of *Trichosporon*. The antifungal therapies initiated either before *Trichosporon* isolation or used for targeted treatment were analyzed. Removal of intravenous catheter was also recorded. The outcome was considered to be favorable if the patient survived or if the infection was considered as cured at day 30 post-diagnosis.

### Antifungal susceptibility testing studies

We selected *in vitro* susceptibility testing studies that included ≥10 isolates for a given species and limited our analysis to those having used a microdilution method. Only drugs with an intravenous formulation were retained. Data on echinocandins susceptibility were not included since *Trichosporon* exhibit natural resistance to these drugs (Espinel-Ingroff, 2003). We also compared antifungal susceptibility profile with the different *Trichosporon asahii* genotypes.

### Statistical analysis

Comparisons between groups were performed using Fisher's exact or Chi-square tests when appropriate for the categorical variables. For continuous non-parametric variables, comparisons were made applying the Mann-Whitney *U*-test and the Kruskal-Wallis one-way analysis of variance test. *P*-values of < 0.05 were considered to be statistically significant. Independent variable found to be associated with death in a univariate analysis with a *p* < 0.2, were included in a binary logistic regression analysis. For this part, the level of statistical significance was set at *p* < 0.05. For each statistically significant factor, an odds ratio (OR) and 95% confidence interval (CI) were computed Stata/IC 13.0 for Mac software (StataCorp, College Station, TX 77845, USA).

## Results

**Figure 1:**
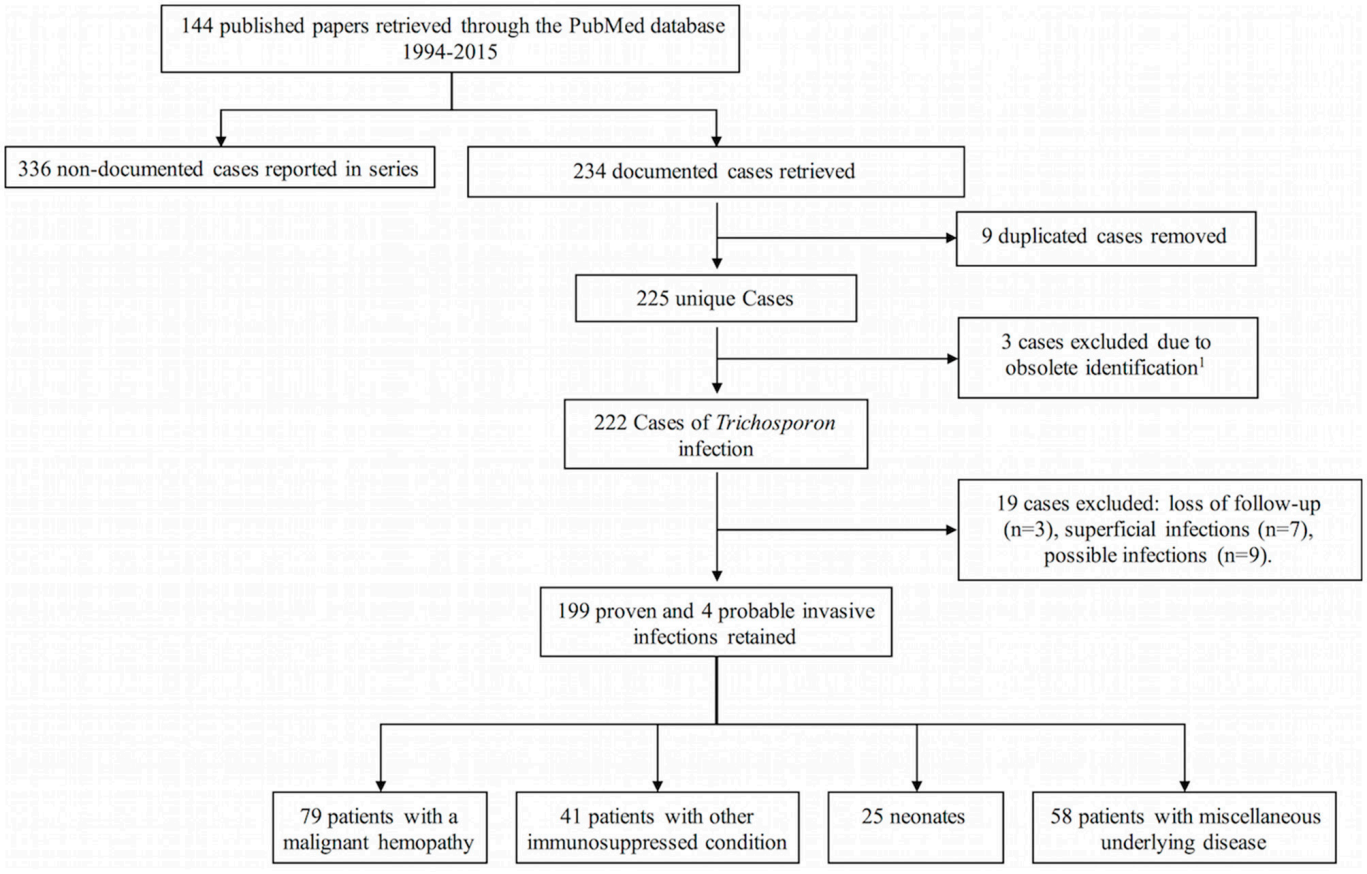
**Flow diagram showing the cases retained after application of selection criteria**. *Trichosporon* pullulans is now considered Gueomyces pullulans.

In a first instance, we found 537 cases of invasive *Trichosporon* infection based on a PubMed search limited between 1994 and 2015. The number of reported cases of invasive *Trichosporon* infections (ITI) grew significantly through the years, from 139 (25.8%) in the 1994–2004 to 398 (74.2%) between the 2005 and 2015 period (Chi-square test; *p* < 0.001). Three hundred and thirty-four cases, mainly recorded in series, were excluded because of insufficient data (Figure 1).

Among the 203 cases retained, 199 cases were proven infection, whereas four were considered probable infection. Excluding newborns (25 cases), the median age of those patients was 41.6 years, but the range varied from 3 days of life to 85 years. The sex ratio was calculated at 0.56.

For comparison analysis, the cases were clustered according to the main underlying disease, e.g., homeopathy (*n* = 79; Grauer et al., 1994; Higgins et al., 1994; Hsiao et al., 1994; Nasu et al., 1994; Hung et al., 1995; Spánik et al., 1995; Itoh et al., 1996; Fanci et al., 1997; Kataoka-Nishimura et al., 1998; Sklair-Levy et al., 1998; Krcmery et al., 1999; Takamura et al., 1999; Erer et al., 2000; Kim et al., 2001; Moretti-Branchini et al., 2001; Fournier et al., 2002; Hadley et al., 2002; Meyer et al., 2002; Chang et al., 2003; Marty et al., 2003; Bassetti et al., 2004; Chowdhary et al., 2004; Viscomi et al., 2004; Antachopoulos et al., 2005; Chan-Tack, 2005; Akagi et al., 2006; Ghiasian et al., 2006; Kendirli et al., 2006; Koyanagi et al., 2006; Matsue et al., 2006; Meguro-Hashimoto et al., 2006; Rodrigues et al., 2006; Hara et al., 2007; Miura et al., 2007; Rieger et al., 2007; Bayramoglu et al., 2008; Hosoki et al., 2008; Thibeault et al., 2008; Tsuji et al., 2008; Fekkar et al., 2009; Walia et al., 2009; Gabriel et al., 2011; Kudo et al., 2011; Hosokawa et al., 2012; Menezes et al., 2012; Chen et al., 2014; Issarachaikul et al., 2014; Karapinar et al., 2014; Capoor et al., 2015; Odero et al., 2015; Pérard et al., 2015; Tanyildiz et al., 2015), other immunodeficiency conditions (*n* = 41; Canales et al., 1998; Lascaux et al., 1998; Anuradha et al., 2000; Piwoz et al., 2000; Ebright et al., 2001; Abliz et al., 2002; Chakrabarti et al., 2002; Kahana et al., 2003; Nettles et al., 2003; Wynne et al., 2004; Abdala et al., 2005; Gunn et al., 2006; Karabay et al., 2006; Rodrigues et al., 2006; Biasoli et al., 2008; David et al., 2008; Gross and Kan, 2008; Chagas-Neto et al., 2009; Lacasse and Cleveland, 2009; Servonnet et al., 2010; Fadhil et al., 2011; Macêdo et al., 2011; Songcharoen et al., 2011; Basiri et al., 2012; Hirschi et al., 2012; Ogura et al., 2012; Tsai et al., 2012; Chen et al., 2013; Almeida Júnior et al., 2014; Yang et al., 2014; Chaitanya et al., 2015; Nobrega de Almeida Júnior et al., 2015; Ozkaya-Parlakay et al., 2016), miscellaneous (*n* = 58; De Saedeleer et al., 1994; Lopes et al., 1994, 1995, 1997; Miralles et al., 1994, p. 10; Miró et al., 1994; Sidarous et al., 1994; Still et al., 1994; Hajjeh and Blumberg, 1995; Mathews and Prabhakar, 1995; Melez et al., 1995; Chaumentin et al., 1996; Kouppari et al., 1997; Wang and Lin, 1999; Cawley et al., 2000; Lo Passo et al., 2001; Mooty et al., 2001; Wolf et al., 2001; Chitra et al., 2002; Kustimur et al., 2002; Reddy et al., 2002; Crowther et al., 2003; Madariaga et al., 2003; Spirn et al., 2003; Ramos et al., 2004; O'Gorman et al., 2006; Rodrigues et al., 2006; Kim et al., 2007, 2008; Tian et al., 2007; Fagundes Júnior et al., 2008; Jian et al., 2008; Chagas-Neto et al., 2009; Hickey et al., 2009; Izumi et al., 2009; Shang et al., 2010; Slocumb et al., 2010; Heslop et al., 2011; Tsai et al., 2011; Liao et al., 2012; Padhi et al., 2014; Gonul et al., 2015; Kumar et al., 2015; Negi et al., 2015; Zuo et al., 2015) and newborns (*n* = 25; Yoss et al., 1997; Sweet and Reid, 1998; Gökahmetoğlu et al., 2002; Panagopoulou et al., 2002; Salazar and Campbell, 2002; Yildiran et al., 2003; Maheshwari et al., 2004; Chagas-Neto et al., 2009; Pereira et al., 2009; Vashishtha et al., 2012; Basu et al., 2015). The main clinical characteristics of each group are summarized in Table 1 and are detailed in Tables S1–S4.

**Table 1:** **Characteristics of 203 cases of invasive *Trichosporon* infection according to the underlying conditions**.

	**Groups of patients**				***p*-value**
	**Hemopathies**	**Other immunodeficiency conditions**	**Newborns**	**Miscellaneous**	
No. of cases (%)	79 (39)	41 (21)	25 (12)	58 (28)	
Age (Mean ± SD)	39.5 ± 21.58	39.3 ± 22.2	NA^a^	46.8 ± 23.1	NS^b^
Sex ratio (F/M)	21/53 0.39	14/23 0.6	8/9 0.8	24/34 0.7	NS
**AT THE TIME OF DIAGNOSIS (%)**					
Neutropenia	67 (85)	3 (8)	0 (0)	0 (0)	**<0.0001**
CVC^c^	36 (46)	15(36)	12 (48)	19 (34)	NS
Breakthrough infection	59 (74)	12 (27)	2 (8)	9(16)	**<0.0001**
Previous antimicrobial therapy	65 (82)	24 (58)	24 (96)	39 (67)	**0.005**
**CLINICAL PRESENTATION**					
Disseminated	79 (100)	25 (61)	25 (100)	33 (56)	**<0.0001**
w/skin lesions	27 (34)	7 (28)	0 (0)	4 (12)	**0.001**
w/pulmonary lesions	33 (42)	6 (24)	1 (5)	3 (9)	**0.0003**
w/liver and/or spleen lesions	11 (14)	1 (4)	0 (0)	1 (3)	**0.06**
Localized deep-seated infections	0 (0)	16 (39)	0 (0)	25 (44)	**<0.0001**
**SPECIES**					
*Trichosporon asahii*^d^	32(40)	20 (48)	16 (64)	27 (46)	NS
*Trichosporon inkin*^e^	1 (1)	9 (22)	0 (0)	6 (11)	**0.0003**
*Trichosporon mucoides/dermatis*^f^^,^ ^g^	2 (2)	4 (9)	3 (12)	2 (3)	NS
Other Species^h^	4 (5)	2 (5)	1 (4)	5 (10)	NS

a NA, Not applicable.

b NS, not statistical.

c Central venous catheter.

d Ten, six, and ten isolates had IGS1, ITS1, and D1/D2 sequence-based identification, respectively.

e Four, three, and three isolates had IGS1, ITS1, and D1/D2 sequence-based identification, respectively.

f The isolates identified as Trichosporon mucoides by phenotypical methods, which do not distinguish T. mucoides from T. dermatis in the absence of IGS1 sequence-based identification, were merged into a single group (Gunn et al., 2006).

g Two and one isolates had IGS1 and ITS1 sequence-based identification, respectively.

h Trichosporon asteroides (n = 5, four and had IGS1 and D1/D2 sequence-based identification, respectively) Trichosporon loubieri (n = 2, ITS1 sequence-based identification), Trichosporon mycotoxinovorans (n = 2, ITS1 and D1/D2 sequence-based identification), Trichosporon laibachii (n = 1, ITS1 sequence-based identification), Trichosporon japonicum (n = 1, ITS1 sequence-based identification), Trichosporon faecale (n = 1, IGS1 sequence-based identification). To delineate the species-specific peculiarities, the reports describing infections attributed to the obsolete T. beigelli and T. cutaneum classifications were considered as caused by Trichosporon sp. Significant differences are indicated in bold.

### Clinical presentation of ITIs

The main clinical presentations of 203 cases of ITI are summarized in Table 1. Among the 199 cases of proven infection, dissemination was reported in 162 cases (79.8%) mainly due to fungemia (*n* = 151, 93.2%). Blood was the unique site of isolation in 90 cases. In other cases, the patients presented mainly with skin (*n* = 13), lungs (*n* = 17), skin and lungs (*n* = 14), liver and/or spleen (*n* = 10), brain (*n* = 4), eyes (*n* = 3) involvement. In eleven patients without fungemia, disseminated trichosporonosis was diagnosed based on the involvement of the skin (*n* = 3), skin and brain (*n* = 3), skin and lungs (*n* = 2), skin and digestive tract (*n* = 1), skin and liver (*n* = 1), heart valve and embolic material/skin (*n* = 1). In the case of proven localized deep-seated infection (*n* = 37), the following organs were predominantly involved: peritoneum (*n* = 14), lower respiratory tract (*n* = 8), brain (*n* = 3), eye (*n* = 3), esophagus (*n* = 3). Finally, four probable *Trichosporon* pulmonary infections were retained.

#### Patients with hematologic disease (n = 79)

Acute myeloid leukemia (*n* = 40; 50.6%) was the most frequent underlying disease in this group of patients, followed by acute lymphoid leukemia (*n* = 17) and myelodysplasic syndrome (*n* = 5). Sixty-seven of those patients (84.8%) were neutropenic at the time of diagnosis of the *Trichosporon* infection. Eight patients (10.1%) developed invasive trichosporonosis after a bone marrow or a blood cord transplant, four (50%) during the pre-engraftment period and under antifungal prophylaxis [(amphotericin B (AMB) *n* = 2, caspofungin *n* = 1, voriconazole *n* = 1)], four (50%) after severe graft-vs.-host disease and under AMB prophylaxis. Thirty-six patients (45.5%) had at least one central venous catheter (CVC) when the diagnosis of ITI was established. All the cases corresponded to a disseminated infection, 29 (36.7%) being isolated fungemia. Organ involvement occurred in 50 patients (63.2%). In these cases, pulmonary infection was the most common (*n* = 33; 41.7%), followed by skin lesions, which were observed in 27 cases (34.1%). On the whole group, an antimicrobial therapy regimen prior to the episode of ITI was mentioned in 65 cases (82.2%). Fifty-nine patients (74.6%) had breakthrough *Trichosporon* infection during treatment with different antifungal drugs, including an echinocandin (*n* = 16), AMB (*n* = 25) or azole derivatives (*n* = 18).

#### Other immunodeficiency disorders (n = 41)

In this group, trichosporonosis mainly occurred in patients with solid organ transplantation (*n* = 13), solid tumors (*n* = 11) or autoimmune diseases (*n* = 8). Kidney transplantation (*n* = 6) and lung cancer (*n* = 4) were the main underlying conditions within the two predominant groups. A disseminated infection was diagnosed in 25 cases (60.9%) and deep-organ involvement was noticed in 12 cases (48%); skin and pulmonary lesions being noticed in 7 and 6 cases, respectively. Isolated pneumonia was diagnosed in three cases, and another three cases had chronic lung infections presenting with pulmonary abscesses or a penetrating chest wall mass. Twenty-four (58.5%), 14 (34.1%), and 13 (31.7%) patients had received antimicrobials, corticosteroids, and immunosuppressive drugs, respectively, before the diagnosis of the invasive fungal infection. Breakthrough infections were documented in 12 cases (29.2%).

#### Other clinical contexts (n = 58)

Finally, we found 58 cases of ITI in patients with miscellaneous underlying conditions. Forty of them (68.9%) had received antimicrobial therapy before the diagnosis of deed-seated trichosporonosis. Among the 33 patients (56.8%) with disseminated disease, 31 (93.9%) had severe baseline disease [(heart failure or pulmonary embolism (*n* = 8; 24.2%), extensive burns (*n* = 5; 15.1%), polytrauma (*n* = 4; 12.1%)] and were in intensive care units; 20 (60%) of them were reported to have a CVC at the time of the infection. Additionally, four (12.1%) had multiple surgeries before developing disseminated trichosporonosis and another four patients developed disseminated trichosporonosis during hemodialysis (12.1%). Moreover, breakthrough infections occurred in nine patients that had disseminated infection (27.2%).

Fourteen patients (24.1%) were diagnosed with deep-seated post-operative infections, notably endocarditis after valve replacement surgery (*n* = 5). Interestingly, the time between the surgery and the occurrence of endocarditis was quite long, ranging from 3 months to 8 years (median 3 years).

Twelve cases of *Trichosporon* peritonitis were recorded, and 11 (91.6%) of those cases complicated the course of continuous ambulatory peritoneal dialysis.

#### Newborns (n = 25)

Twenty-five cases of ITI occurred in newborns. It is worth noting that eleven cases were reported in two outbreaks from Indian hospitals (Vashishtha et al., 2012; Basu et al., 2015). Infections occurred between the third and the 84th day of life. All of the infants except two were preterm at delivery, and 10 (40%) had extremely low birth weight (< 1000 g). A previous diagnosis of perinatal asphyxia or respiratory distress syndrome was identified in 15 cases (60%), and all of the newborns but one received previous antimicrobial therapy. *Trichosporon* infection was disseminated in all cases either as isolated fungemia (*n* = 22; 88%) or associated with a deep-organ involvement (*n* = 3; 12%). Twelve infants (48%) had a CVC at the time of diagnosis of the ITI.

### Diagnostic procedures

The diagnosis of ITI relied in all the cases on a positive culture for *Trichosporon* from a clinical sample. In the 47 cases with a pathological examination of biopsy specimens, the presence of arthroconidia was only mentioned in eight cases (17%). Positive blood culture(s), as unique fungal isolation, was the most common presentation (*n* = 100; 49.2%). In 69 cases (33.9%), there was no identification at the species level. Species identification relied on molecular analysis of intergenic spacer IGS1 (*n* = 22), ITS1 (*n* = 15) or D1/D2 domain of the 26S regions of the rRNA gene (*n* = 15) (Table 2). In other cases, there was either no information regarding the method used for identification or the isolates were identified using a commercial phenotypic method. Overall, *Trichosporon asahii* (*n* = 95; 46.7%) was the main etiologic agent. Nine additional species were identified: *Trichosporon inkin* (*n* = 16), *Trichosporon dermatis/mucoides* (*n* = 11), and *Trichosporon* spp. (*n* = 12). *T. inkin* was significantly less prevalent in patients with homeopathy than in the rest of the population analyzed (*p* = 0.0003).

**Table 2:** **Outcome predictors in patients with invasive *Trichosporon* infection according to univariate and multivariate analysis**.

			***p*-value**		
	**No. of patients**	**Unfavorable outcome rate (%)**	**Univariate analysis**	**Multivariate analysis**	**Odds Ratio (95% CI)**
Sex			NS		
Female	66	42.42			
Male	120	45.83			
Age			0.042	0.08	1.02 (1.00–1.03)
Underlying disease^a^				NS	
1	79	53.16		NS	
2	41	36.59	0.087^a^		
3	58	34.48	0.031^a^		
4	25	52.00	NS		
Species^a^				NS	
1	95	47.37		NS	
2	15	31.25	NS		
3	11	18.18	0.084^a^		
4	81	46.91	NS		
Breakthrough infection			0.053	0.049	2.45 (1.05–5.97)
Yes	84	52.38			
No	119	38.66			
Disseminated infection			0.001	0.070	2.62 (0.92–7.45)
Yes	162	50.62			
No	41	19.51			
Neutropenia			0.019	NS	
Yes	70	55.71			
No	133	38.35			
Coinfection			NS		
Yes	19	47.37			
No	184	44.02			
Antifungal therapy			0.130	NS	
Amphotericin-based					
Yes	112	49.11			
No	91	38.46			
Echinocandin-based			0.073	NS	
Yes	13	69.23			
No	190	42.63			
Azole based			<0.001	<0.001	0.15 (0.06–0.42)^b^
Yes	101	28.71			
No	102	67.78			
Voriconazole based			0.035	NS	
Yes	33	27.27			
No	170	47.65			

a Comparison to the reference i.e., Trichosporon asahii for species and hematological patients for group of patients, respectively.

b The odds ratio <1 is indicative of protection associated with the used of azole based therapy. Underlying disease: (1) Hemopathies, (2) other immunodeficiency conditions, (3) miscellaneous diseases, (4) neonates. Species: (1) Trichosporon asahii, (2) Trichosporon inkin, (3) Trichosporon dermatis/mucoides, (4) Trichosporon beigelii and other species.

A positive serum *Cryptococcus* glucuronoxylomannan (GXM) antigen assay was reported in 4 of 15 cases (26.6%) while β-D-glucan (BDG) was detected in the serum of 9 among 11 tested patients (81.8%).

### Treatment and outcome

Twenty-five different antifungal regimens were reported. AMB was used in 111 cases, mainly as deoxycholate AMB (*n* = 89) and liposomal AMB (*n* = 18). Thirteen patients received targeted treatment with echinocandins either in monotherapy (*n* = 5) or combined with other antifungal (*n* = 8). All those patients treated with echinocandin monotherapy for disseminated infection died. Fluconazole was the azole drug the most frequently used for the treatment of those infections (*n* = 54). Combined antifungal therapy was commonly used (*n* = 48; 24.2%). Twelve patients did not receive any antifungal treatment, and 11 died. The unfavorable outcome rate was calculated at 44.3%. Table 2 summarizes the impact of various prognosis factors. In the univariate analysis, advanced age, disseminated infection and neutropenia at the time of diagnosis were associated with unfavorable outcome (*p* < 0.05). There was also a similar trend for breakthrough infection and curative therapy including an echinocandin drug (*p* < 0.1). By the contrary, patients from group 2 and 3, and the use of either azole or voriconazole in the therapeutic management were associated with a better outcome. In the multivariate analysis, breakthrough infection and azole-based therapy remained significant (*p* < 0.05) with odds ratio (IC95%) at 2.45 (1.05–5.97) and 0.17 (0.060–0.423), respectively. In neutropenic patients, we also found the recovery of a normal neutrophils count as a favorable prognosis factor with mortality rate at 15 vs. 92.31% (*p* = 0.001). Finally, in patients with hematologic disease, the use of voriconazole in the curative regimen led to a significantly better prognosis: favorable outcome rate at 73.6 vs. 41.8% (*p* = 0.016).

### Antifungal susceptibility testing (AST)

Details from 19 studies having tested at least 10 *Trichosporon* clinical isolates for a given species with a microdilution method are presented in Tables 3–**8** (Arikan and Hasçelik, 2002; Paphitou et al., 2002; Ramos et al., 2004; Metin et al., 2005; Rodriguez-Tudela et al., 2005; de Oliveira Silva et al., 2008; Chagas-Neto et al., 2009; Taj-Aldeen et al., 2009; Thompson et al., 2009; Lemes et al., 2010; Mekha et al., 2010; Guo et al., 2011; Sun et al., 2012; Tsai et al., 2012; Hazirolan et al., 2013; Yang et al., 2013; Arabatzis et al., 2014; Taverna et al., 2014; Montoya et al., 2015). Up to now, there is no recommendation from the two main consortia (EUCAST and CLSI) regarding the AST of *Trichosporon*. Thus, it is important to note that the analyzed studies included some variants in the application of the EUCAST or CLSI method: three studies applied the EUCAST methodology including agitation at 350 rpm during incubation (Rodriguez-Tudela et al., 2005; de Oliveira Silva et al., 2008; Taverna et al., 2014), and one of them incubated the plates at 30°C (Rodriguez-Tudela et al., 2005). Two studies analyzed the CLSI method with MIC determination by spectrophotometric reading (Taj-Aldeen et al., 2009; Tsai et al., 2012). Interestingly, some works included comparisons of either culture media (Tsai et al., 2012) or incubation times (24 vs. 48 h; Arikan and Hasçelik, 2002; Metin et al., 2005; Chagas-Neto et al., 2009; Lemes et al., 2010; Tsai et al., 2012; Hazirolan et al., 2013).

**Table 3:** ***In vitro* susceptibility testing of amphotericin B against *Trichosporon asahii* clinical isolates**.

**(Author, year)**	**No. of tested isolates**	**Country of isolation**	**Method**	**Modifications**	**Incubation time (h)**	**MIC^a^ range (mg/L)**	**MIC 50 (mg/L)**	**MIC 90 (mg/L)**	**GM^b^ (mg/L)**
Arikan and Hasçelik, 2002	43	Turkey	CLSI^c^	No	24	0.5–4	2	4	ND^d^
					48	1–8	4	4	ND
Paphitou et al., 2002	24	USA	CLSI	No	24–48	0.25–8	0.5	ND	ND
Rodriguez-Tudela et al., 2005	15^*^	Spain, Argentine	EUCAST^e^	Agitation at 350 rpm, 30°C incubation	48	2–16	ND	ND	5.2
Metin et al., 2005	13	Turkey	CLSI	No	24	0.03–2	0.06	1	ND
					48	0.03–0.06	0.03	0.06	ND
de Oliveira Silva et al., 2008	10^*^	Brazil	EUCAST	Agitation at 350 rpm	48	2–4.0	2	4	2.4
Chagas-Neto et al., 2009	15^*^	Brazil	CLSI	No	24	0.5–2	ND	ND	0.9
					48	0.5–4	ND	ND	1.4
Thompson et al., 2009	40	USA	CLSI	No	72	0.125–8	0.5	2	0.5
Taj-Aldeen et al., 2009	15^*^	Qatar	CLSI	MIC reading with spectrophotometer	48	2–≥16	8	≥16	ND
Lemes et al., 2010	26	Brazil	CLSI	No	24	0.006–64	2	64	ND
					48	0.006–64	4	64	ND
Mekha et al., 2010	101^*^	Thailand	CLSI	No	24–48	0.125–16	0.5	2	0.7
Guo et al., 2011	35	China	CLSI	No	48	0.03–1	1	1	0.8
Sun et al., 2012	12^*^	China	CLSI	MIC reading with spectrophotometer	24–48	0.25–1	ND	ND	0.5
Tsai et al., 2012	22^*^	China	CLSI	MIC reading with spectrophotometer	24	0.03–1	0.5	1	0.3
					48	0.125–1	0.25	1	0.3
			CLSI	RPMI 2% glucose, MIC reading with spectrophotometer	24	0.25–1	0.5	1	0.5
					48	0.5–16	1	2	1.17
			EUCAST	No	24–48	0.25–2	0.75	1	0.7
Yang et al., 2013	32^*^	China	CLSI	No	48	≤0.5–4	0.5	2	ND
Iturrieta-González et al., 2014	20	Brazil	CLSI	No	24–48	1–16	4	4	2.5
Arabatzis et al., 2014	37^*^	Australia	CLSI	No	24–48	0.03–64	2	32	1.9
			EUCAST	No	24–48	0.06–32	2	16	1.9
Taverna et al., 2014	29^*^	Argentina	EUCAST	No	24–48	0.25–4	ND	ND	0.9
Montoya et al., 2015		Mexico	CLSI	No	24	0.5–16	2	4	1.84

a MIC, minimal inhibitory concentration.

b GM, Geometric mean.

c CLSI, Clinical Laboratory Standards Institute.

d ND, not described.

e EUCAST, European Committee on Antimicrobial Susceptibility Testing.

* Studies having confirmed species identification using molecular analysis.

#### AST of Trichosporon asahii isolates

By far, *T. asahii* was the most studied species (636 isolates evaluated amongst 698). The distribution of minimal inhibitory concentrations (MICs) against AMB was quite heterogeneous (Table 3), with MIC_50_s (concentration that inhibits the growth of 50% of the tested isolates), MIC_90_s (concentration that inhibits the growth of 90% of the tested isolates) and MIC geometric means (GMs) ranging between 0.03–8, 1–64, and 0.26–5.2 mg/L, respectively. Higher MIC values for AMB were described notably in two studies that applied the EUCAST methodology with agitation during incubation (isolates from Spain, Argentina, and Brazil; Rodriguez-Tudela et al., 2005; de Oliveira Silva et al., 2008).

Among the azole compounds, fluconazole was the compound most extensively analyzed (Table 4). MIC distribution of fluconazole was also heterogeneous, with MIC_50_s, MIC_90_s and MIC geometric means (GMs) ranging between 0.5–16, 1–64, and 0.8–17.1 mg/L, respectively. Again, higher MIC values were described notably in three studies that applied the EUCAST methodology with agitation during incubation (isolates from Spain, Argentina, and Brazil; Rodriguez-Tudela et al., 2005; de Oliveira Silva et al., 2008; Taverna et al., 2014). Lowest MICs were observed for the last generation of triazoles, i.e., voriconazole (Table 5), and indeed for isavuconazole and posaconazole (Table 6).

**Table 4:** ***In vitro* susceptibility testing of fluconazole against *Trichosporon asahii* clinical isolates**.

**(Author, year)**	**No. of tested isolates**	**Country of isolation**	**Method**	**Modifications**	**Incubation time (h)**	**MIC^a^ range (mg/L)**	**MIC 50 (mg/L)**	**MIC 90 (mg/L)**	**GM^b^ (mg/L)**
Arikan and Hasçelik, 2002	43	Turkey	CLSI^c^	No	24	0.5–4	1	4	ND^d^
					48	1–8	2	8	ND
Paphitou et al., 2002	24	USA	CLSI	No	24–48	0.5–>64	2	ND	ND
Rodriguez-Tudela et al., 2005	15^*^	Spain, Argentine	EUCAST^e^	Agitation at 350 rpm, 30°C incubation	48	0.5–64	ND	ND	7.6
Metin et al., 2005	13	Turkey	CLSI	No	24	0.5–1	0.25	1	ND
					48	1–2	0.5	2	ND
de Oliveira Silva et al., 2008	10^*^	Brazil	EUCAST	Agitation at 350 rpm	48	16–32	16	32	17.1
Chagas-Neto et al., 2009	15^*^	Brazil	CLSI	No	24	0.25–2	ND	ND	0.8
					48	0.25–8	ND	ND	1.3
Thompson et al., 2009	40	USA	CLSI	No	72	0.25–>64	1	2	1.2
Taj-Aldeen et al., 2009	15^*^	Qatar	CLSI	MIC reading with spectrophotometer	48	0.25–64	4	8	ND
Lemes et al., 2010	26	Brazil	CLSI	No	24	0.5–8	2	8	ND
					48	0.5–16	4	8	ND
Mekha et al., 2010	101^*^	Thailand	CLSI	No	24–48	4–64	8	64	9.6
Guo et al., 2011	35	China	CLSI	No	48	1–32	2	8	2
Sun et al., 2012	12^*^	China	CLSI	MIC reading with spectrophotometer	24–48	0.5–4	ND	ND	1.2
Tsai et al., 2012	22^*^	China	CLSI	MIC reading with spectrophotometer	24	0.125–16	1	4	1.3
					48	0.125–16	2	4	1.7
			CLSI	RPMI 2% glucose, MIC reading with spectrophotometer	24	0.125–16	1	2	1
					48	0.5–16	2	4	2.2
			EUCAST	No	24–48	0.125–4	1	4	1.2
Yang et al., 2013	32^*^	China	CLSI	No	48	≤2–16	2	4	ND
Hazirolan et al., 2013	90	Turkey	CLSI	No	24	0.125–8	1	4	1.5
					48	0.5–16	4	8	2.2
Iturrieta-González et al., 2014	20	Brazil	CLSI	No	24–48	2–4	4	4	3.1
Arabatzis et al., 2014	37^*^	Australia	CLSI	No	24–48	1.0–64	8	64	8.5
			EUCAST	No	24–48	0.5–64	8	64	7.8
Taverna et al., 2014	29^*^	Argentina	EUCAST	No	24–48	1–64	ND	ND	7.63
Montoya et al., 2015		Mexico	CLSI	No	48	0.125–16	0.5	1	0.78

a MIC, minimal inhibitory concentration.

b GM, Geometric mean.

c CLSI, Clinical Laboratory Standards Institute.

d ND, not described.

e EUCAST, European Committee on Antimicrobial Susceptibility Testing.

* Studies having confirmed species identification using molecular analysis.

**Table 5:** ***In vitro* susceptibility testing of voriconazole against *Trichosporon asahii* clinical isolates**.

**(Author, year)**	**No. of tested isolates**	**Country of isolation**	**Method**	**Modifications**	**Incubation time (h)**	**MIC^a^ range (mg/L)**	**MIC 50 (mg/L)**	**MIC 90 (mg/L)**	**GM^b^ (mg/L)**
Rodriguez-Tudela et al., 2005	15*	Spain, Argentine	EUCAST^c^	Agitation at 350 rpm, 30°C incubation	48	0.03–8	ND^d^	ND	0.3
Chagas-Neto et al., 2009	15^*^	Brazil	CLSI^e^	No	24	0.03–0.06	ND	ND	0.03
					48	0.03–0.06	ND	ND	0.03
Thompson et al., 2009	40	USA	CLSI	No	72	0.03–0.12	0.03	0.06	0.03
Taj-Aldeen et al., 2009	15^*^	Qatar	CLSI	MIC reading with spectrophotometer	48	0.016–2	0.12	0.25	ND
Lemes et al., 2010	26	Brazil	CLSI	No	24	0.03–4	0.5	1	ND
					48	0.03–4	1	2	ND
Mekha et al., 2010	101^*^	Thailand	CLSI	No	24–48	0.06–0.25	0.12	0.25	0.09
Guo et al., 2011	35	China	CLSI	No	48	0.03–1	0.06	0.5	0.08
Sun et al., 2012	12^*^	China	CLSI	MIC reading with spectrophotometer	24–48	0.03–0.12	ND	ND	0.06
Tsai et al., 2012	22^*^	China	CLSI	MIC reading with spectrophotometer	24	0.016–2	0.06	0.5	0.03
					48	0.016–8	0.03	0.06	0.03
			CLSI	RPMI 2% glucose, MIC reading with spectrophotometer	24	0.125–4	0.5	2	0.5
					48	0.25–8	0.5	4	0.6
			EUCAST	No	24–48	0.125–1	0.5	1	0.4
Yang et al., 2013	32^*^	China	CLSI	No	48	≤ 0.03–8	0.03	0.06	ND
Arabatzis et al., 2014	37^*^	Australia	CLSI	No	24–48	0.064–32	1	32	1.07
			EUCAST	No	24–48	0.064–32	1	32	1.06
Taverna et al., 2014	29^*^	Argentina	EUCAST	No	24–48	0.03–0.5	ND	ND	0.12
Iturrieta-González et al., 2014	20	Brazil	CLSI	No	24–48	0.03–0.06	0.03	0.06	0.04
Montoya et al., 2015		Mexico	CLSI	No	48	0.03–1	0.03	0.03	0.04

a MIC, minimal inhibitory concentration.

b GM, Geometric mean.

c EUCAST, European Committee on Antimicrobial Susceptibility Testing.

d ND, not described.

e CLSI, Clinical Laboratory Standards Institute.

* Studies having confirmed species identification using molecular analysis.

**Table 6:** ***In vitro* susceptibility testing of posaconazole and isavuconazole against *Trichosporon asahii* clinical isolates**.

**(Author, year)**	**No. of tested isolates**	**Country of isolation**	**Method**	**Antifungal**	**Incubation time**	**MIC^a^ range (mg/L)**	**MIC 50 (mg/L)**	**MIC 90 (mg/L)**	**GM^b^ (mg/L)**
Paphitou et al., 2002	24	USA	CLSI^c^	Posaconazole	24–48	0.06–>16	0.125	ND^d^	ND
Thompson et al., 2009	40	USA	CLSI	Posaconazole	72	0.06–0.5	0.25	0.25	0.2
Taj-Aldeen et al., 2009	15^*^	Qatar	CLSI, MIC reading with spectrophotometer	Posacanazole	48	0.06–0.25	0.25	0.25	ND
				Isavuconazole	48	0.008–0.5	0.125	0.125	ND
Hazirolan et al., 2013	90	Turkey	CLSI	Posaconazole	24	≤ 0.015–1	0.125	0.5	0.1
					48	0.06–1	0.25	0.5	0.2
				Isavuconazole	24	≤ 0.015–0.5	0.03	0.25	0.07
					48	≤ 0.015–0.5	0.125	0.25	0.1
Arabatzis et al., 2014	37^*^	Australia	CLSI	Posaconazole	24–48	0.03–16	1	4	0.9
			EUCAST^e^	Posacanazole	24–48	0.06–32	1	4	1.4
Taverna et al., 2014	29^*^	Argentina	EUCAST	Posaconazole	24–48	0.015–1	ND	ND	0.2
Montoya et al., 2015	39	Mexico	CLSI	Posaconazole	48	0.03–0.5	0.06	0.25	0.08

a MIC, minimal inhibitory concentration.

b GM, Geometric mean.

c CLSI, Clinical Laboratory Standards Institute.

d ND, not described.

e EUCAST, European Committee on Antimicrobial Susceptibility Testing.

* Studies having confirmed species identification using molecular analysis.

Interestingly, while individual studies did not find any correlation between genotype and *in vitro* susceptibility, when merging the data available from 4 well-designed studies (Rodriguez-Tudela et al., 2007; Guo et al., 2011; Yang et al., 2013; Arabatzis et al., 2014), we noted that genotype 3 strains may exhibit higher MICs to voriconazole than genotype 1 strains: MIC GM 0.12 vs. 0.49 mg/L, *p* = 0.04, whereas the MIC values of AMB (MIC GM 1.9 vs. 1.2, *p* = 0.2) and fluconazole (MIC GM 5.2 vs. 5.6, *p* = 0.7) were similar for both genotypes.

Of note, the reading at 48 h instead of 24 h only slightly increases the MICS by one dilution and the results obtained either using the CLSI or the EUCAST method are quite similar with the possible exception of voriconazole for which higher MICs were observed using the EUCAST method in one of two studies (Tsai et al., 2012; Arabatzis et al., 2014).

#### AST of Trichosporon inkin, Trichosporon mucoides/dermatis isolates

In comparison to *T. asahii*, a much narrow MICs distribution for AMB was described for both species, with MIC GM ranging between 0.21–0.29 mg/L for *T. inkin* (Table 7) and 0.61–0.69 mg/L for *T. mucoides/dermatis* (Table 8). Conversely, a wide range of MIC GMs of fluconazole against *T. mucoides/dermatis* has been reported, ranging from 0.25 to 7 mg/L. Two of three studies described higher fluconazole MICs of *T. mucoides/dermatis* isolates in comparison to the MICs of *T. asahii* isolates (Metin et al., 2005; Rodriguez-Tudela et al., 2005). The MIC GM of fluconazole against *T. inkin* isolates ranged from 2 to 2.74 mg/L. Rodriguez-Tudella and collaborators described lower MICs of fluconazole against *T. inkin* isolates in comparison to *T. asahii* and *T. mucoides/dermatis* isolates (Rodriguez-Tudela et al., 2005). Voriconazole exhibited the lowest MICs against both *T. inkin* and *T. mucoides/dermatis*, with MIC GMs ranging from 0.11 to 0.12 for *T. inkin* and 0.03 to 0.25 for *T. mucoides/dermatis.*

**Table 7:** ***In vitro* susceptibility testing against *Trichosporon inkin* clinical isolates**.

**(Author, year)**	**No. of tested isolates**	**Country of isolation**	**Method**	**Incubation time (h)**	**Antifungal**	**MIC^a^ range (mg/L)**	**MIC 50 (mg/L)**	**MIC 90 (mg/L)**	**GM^b^ (mg/L)**
Ramos et al., 2004	11	Spain	EUCAST^c^, Agitation at 350 rpm, 30°C incubation	48	AMB^d^	0.006–1	0.25	1	0.3
					Fluconazole	1.0–32	2	8	2.7
					Voriconazole	0.03–0.5	0.12	0.5	0.11
Rodriguez-Tudela et al., 2005	11^*^	Spain, Argentine	EUCAST, Agitation at 350 rpm, 30°C incubation	48	AMB	0.03–1	ND	ND	0.2
					Fluconazole	0.5–4	ND	ND	2
					Voriconazole	0.03–2	ND	ND	0.12

a MIC, minimal inhibitory concentration.

b GM, Geometric mean.

c EUCAST = European Committee on Antimicrobial Susceptibility Testing.

d AMB, amphotericin B.

* Studies having confirmed species identification using molecular analysis.

**Table 8:** ***In vitro* susceptibility testing against *Trichosporon mucoides/dermatis* clinical isolates**.

**(Author, year)**	**No. of tested isolates**	**Country of isolation**	**Method**	**Antifungal**	**Incubation time (h)**	**MIC^a^ range (mg/L)**	**MIC 50 (mg/L)**	**MIC 90 (mg/L)**	**GM^b^ (mg/L)**
Rodriguez-Tudela et al., 2005	16^*^	Spain, Argentine	EUCAST^c^, Agitation at 350 rpm, 30°C incubation	AMB^d^	48	0.03–1	ND	ND	0.2
				Fluconazole	48	0.5–4	ND	ND	2
				Voriconazole	48	0.03–2	ND	ND	0.12
Metin et al., 2005	14	Turkey	CLSI^e^	AMB	24	0.03–4	0.03	0.25	ND
					48	0.03–4	0.06	2	ND
				Fluconazole	24	0.125–32	1	2	ND
					48	0.125–64	2	8	ND
				Voriconazole	24	0.03–0.5	0.03	0.03	ND
					48	0.3–0.5	0.03	0.25	ND
Thompson et al., 2009	10	USA	CLSI	AMB	72	0.125–8	0.5	2	0.5
				Fluconazole	72	0.12–1	0.12	1	0.25
				Voriconazole	72	0.03–0.06	0.03	0.06	0.03

a MIC, minimal inhibitory concentration.

b GM, Geometric mean.

c EUCAST, European Committee on Antimicrobial Susceptibility Testing.

d AMB, amphotericin B.

e CLSI, Clinical and Laboratory Standards Institute.

* Studies having confirmed species identification using molecular analysis.

## Discussion

*Trichosporon* emerged as a pathogen in the 70–80's, mainly for neutropenic patients (Gold et al., 1981; Walsh et al., 1986). Despite the lack of data providing the real incidence of ITI at this time, published series supported the fact that *Trichosporon* was the second most frequent agent responsible for fungemia in this population (Leblond et al., 1986; Anaissie et al., 1992). Based on clinical and experimental data, it was then shown that azoles had a significant activity against *Trichosporon*, while being less toxic that the standard at that time treatment, amphotericin B (Anaissie et al., 1992). This and the wider use of azole drugs either as prophylaxis or empirical therapy probably explain the decreasing concern regarding ITI in 90's. Between 1989 and 2002, an estimated incidence, of 81 cases per 100,000 admissions and 3 per 100,000 admissions were reported in patients with hematologic malignancies and patients with solid tumors, respectively (Kontoyiannis et al., 2004). Similarly, in an Italian multicentric study conducted in 15 hematology departments between 1983 and 2002, an incidence of 0.4% was observed in patients with acute leukemia (Girmenia et al., 2005). However, more recently, the MD Anderson Cancer Center (TX, USA) demonstrated an increase in the incidence of invasive yeast infections not due to *Candida* or *Cryptococcus*, of which 20% were *Trichosporon*, from 1.8 to 2.35 cases per 100,000 patient-days between the periods 1998–2004 and 2005–2010 (Chitasombat et al., 2012). Again this may reflect the change in the antifungal stewardship for neutropenic patients. Indeed, echinocandin drugs, which emerged as valuable options of choice for either prophylaxis, pre-emptive or empirical therapy, have no effect on *Trichosporon* (Asada et al., 2006; Matsue et al., 2006; Hiramatsu et al., 2008; Suzuki et al., 2010; Park et al., 2014; Nachbaur et al., 2015). Our review supports this re-emergence with a significant increase of reported cases in the second part of the 2000s decade.

In accordance with previous studies, we found that hematological diseases (38.9% of the cases), mainly acute leukemia, remain the most common underlying condition of ITI, however, with a much smaller percentage than previous reported (Girmenia et al., 2005). Eighty-five percent of the patients with hematological diseases were neutropenic at the time of ITI diagnosis, supporting the fact that neutrophil cells are, as for candidiasis and aspergillosis, essential in the prevention of ITI. The disruption of the mucosal barrier owing to chemotherapy-induced mucositis may also contribute to invasion of the yeast cells. This, combined with the modification of the digestive flora due to previous antibiotic therapies, observed in 58–96% of the cases according to the patients' group, may favor the translocation of yeasts from the digestive tract to the blood vessels. However, our study also revealed that not only patients with hematological malignancy are now concerned, as we showed that other conditions mainly related to severe baseline diseases (e.g., SOT, auto-immune diseases, solid tumor, preterm birth, extensive skin lesions such as burns, or pemphigus) and invasive procedures (e.g., CVC, prosthetic heart valves), may also favor the occurrence of ITI.

The origin of *Trichosporon* infections, notably post-operative ones, is uncertain. Indeed, *Trichosporon* has been isolated from the hospital environment (Fanfair et al., 2013), but is also considered a commensal of the digestive tract and may colonize the skin of healthy patients (Colombo et al., 2011; Zhang et al., 2011; Cho et al., 2015). In neonates, it is likely that the increasing permissiveness of the natural barriers, mainly the skin and the digestive mucosa, may favor the translocation of any yeast types, including *Trichosporon*, which has also been isolated from the skin of preterm infants (Kaufman et al., 2001). In addition, total parenteral nutrition, frequently used in this context, may have been the source of an outbreak of *T. asahii* bloodstream infections (Vashishtha et al., 2012).

In any case, the clinical features of invasive trichosporonosis were not specific. The persistence of fever during ongoing treatment with AMB or echinocandins may be more supportive (Colombo et al., 2011). Dissemination is a common feature, particularly in patients with homeopathy and newborns (100% of the cases). While difficult to interpret when solely based on culture results, pulmonary lesions are frequently reported as diffuse nodules, lobar pneumonia, reticulonodular infiltrates, or mass-like lesions (Akagi et al., 2006; Kendirli et al., 2006). Positive culture for *Trichosporon* from respiratory tract samples, notably when repeated should always be interpreted with caution in patients with risk factors. Skin lesions were the second most common tissue involvement and warrant the addition of trichosporonosis to the list, which included candidiasis and fusariosis, as a cause of skin lesions during febrile neutropenia. The cutaneous lesions, mainly located in the lower extremities, the trunk and the face, are typically hemorrhagic macular or maculopapular, and differ in subtle way from those of *Candida* that tend to be punctate, pseudopustular (necrotic epicenter) with a rim of erythema (Nucci et al., 1992; Higgins et al., 1994; Miura et al., 2007).

The diagnosis of ITI always relies on the isolation of a yeast-like organism from a clinical specimen further identified as *Trichosporon*. Direct examination of clinical specimens rarely contributed to diagnosis as it rarely demonstrated arthroconidia in samples. It is remarkable than about a third of the isolates had not been identified at the species level. Phenotypic approaches have been shown to be poorly efficient and direct sequencing of the IGS1 region of the ribosomal DNA is now considered the reference method for species identification of *Trichosporon* isolates (Sugita et al., 2002). MALDI-TOF mass spectrometry, which has recently been shown to be a valuable alternative for routine identification (de Almeida Júnior et al., 2014), should help at reducing the part of non- or uncertainly-identified isolates in order to test more in depth, possible species-specific epidemiological traits, and clinical scenarios. Therapeutic adaptation may also be required according to the causative species.

Unfortunately, serum markers are either unsatisfactory or have not been evaluated in depth for the diagnosis of ITI. Although, it is well-known that *Trichosporon* shares some antigenic properties with *Cryptococcus neoformans* (Campbell et al., 1985), a cross reaction with the cryptococcal GXM antigen assay was only found in 26% of the tested cases; a negative assay thus does not rule out the diagnosis. BGD assays may be more interesting with nine positive results among 11 sera tested. Similarly, in a case series study, 12 out of 25 patients with *Trichosporon* fungemia have been reported with significant levels of BDG (Suzuki et al., 2010). While the use of molecular tools, not commercially available, is more anecdotal, a study reported a good performance of a real-time PCR assay to detect *T. asahii* DNA from the blood samples (Tsuji et al., 2008).

*T. asahii* was shown to be associated with a poor prognosis what may be due in part to its lower susceptibility to antifungal drugs (Pfaller et al., 2009). Nevertheless, neither the CLSI nor the EUCAST consortium has proposed a standardized procedure for AST of *Trichosporon*. Zaragoza et al. analyzed different parameters for optimal growth of nonfermentative yeasts, including some *Trichosporon* strains, for AST (Zaragoza et al., 2011), and showed that the use of yeast nitrogen base medium (YNB), agitation and incubation at 30°C resulted in a better growth and a more reliable and stable measurement of the MIC values. However, whatever was the conditions used they found an excellent agreement between the methods when reading the MICs after 48 h of incubation (Zaragoza et al., 2011).

Despite the lack of defined clinical breakpoints, the constantly elevated MICs against the echinocandins support natural resistance to this antifungal class (Colombo et al., 2011). Similarly, because the minimal fungicidal concentrations of polyenes have been shown to be 20-fold greater than the minimal inhibitory concentration, the *Trichosporon* genus was also considered naturally resistant to these drugs, notably AMB (Walsh et al., 1990). However, after the taxonomic reevaluation, it has been shown that *T. asahii* clearly exhibits higher MICs against AMB compared to other species, such as *T. mucoides/dermatis* and *T. inkin* (Rodriguez-Tudela et al., 2005; Tsai et al., 2012). On the contrary, *T. mucoides/dermatis* appears to be more resistant *in vitro* to fluconazole compared to other species (Metin et al., 2005; Rodriguez-Tudela et al., 2005). Nevertheless, whatever is the species considered, voriconazole, posaconazole, and isavuconazole offer a better *in vitro* efficacy compared to fluconazole, even if some *T. asahii* isolates belonging to genotype 3 with reduced susceptibility to voriconazole has been described (Yang et al., 2013; Arabatzis et al., 2014). The heterogeneity of population structure of *T. asahii* may also explain the great variability in MICs found in this species against the different antifungals evaluated.

To the best of our knowledge, there is no study designed specifically to compare different antifungal regimens in the management of those infections. The lack of antifungal treatment led to a fatal outcome almost constantly. In our review, the overall unfavorable outcome rate was at 44.3%. This rate was quite similar to that reported in a US Cancer Center (50%) but slightly lower than that found in the study by Girmenia et al. (64.7%), where AMB was the main (76.5%) drug used for treatment. Indeed, our review suggests that azole-based therapy may be superior to echinocandin- or amphotericin-based therapies. Similar findings were reported in a retrospective study of 33 patients with hematological malignancy: the mortality rate was 63% with azole-based therapy vs. 100% in the absence of any azole drug in the therapeutic regimen (*p* = 0.031; Suzuki et al., 2010). In our study, the use of voriconazole significantly improved the prognosis of patients with hematological malignancy supporting the recent recommendations proposed by the ESCMID (Arendrup et al., 2014).

## Conclusions

Although, hematologic malignancies are the main underlying diseases, invasive *Trichosporon* infection may also occur in other contexts of immunosuppression, in newborns and in various conditions of debilitating diseases, with different clinical presentations. Due to the natural resistance to echinocandins and polyenes of *Trichosporon*, breakthrough infections are common. Bloodstream infection sometimes combined with pneumonia and/or skin lesions are the most common clinical feature in patients with hematologic malignancies. *T. asahii* is the predominant causative species and is associated with a poor prognosis, possibly linked to its reduce sensitivity to some azole drugs. Nevertheless, first line therapy should rely on azole derivatives and particularly voriconazole, which exhibits the best *in vitro* activity against *Trichosporon* species and significantly leads to a better outcome in patients with underlying homeopathy.

## Author contributions

JN: designed the study, helped with acquisition and data analysis, drafted and revised the work, approved the final work and agrees with all the aspects of the work; CH: designed the study, helped with data analysis, drafted and revised the work, approved the final work, and agree with all the aspects of the work.

### Conflict of interest statement

JN was supported by grants from Fundação de Amparo à Pesquisa do Estado de São Paulo (2011/08911-0). CH received a grant from Pfizer for the development of a MALDI-TOF approach for the identification of Trichosporon clinical isolates. CH received fees for oral speaker from Astellas and Merk Sharp and Dohme. CH received travel grants from Pfizer, Astellas, and Merk Sharp and Dohme.
