# Supplementary material for: Invasive Trichosporon Infection: a Systematic Review on a Re-emerging Fungal Pathogen
Source: Front Microbiol. 2016 Oct 17;7:1629. doi: 10.3389/fmicb.2016.01629 (PMC5065970; doi:10.3389/fmicb.2016.01629)
Supplement: Supplementary file 1 [file Table1.DOCX]

**Table S1.** Summary of the seventy-nine cases of invasive trichosporonosis in patients with hemopathies.

| Ref. | Age/  Sex | Species | First isolate | Other sites of infection | Serological tests | Concomitant  organisms | Baseline  disease | N<500^1^ | ATB^2^ | CVC^3^ |
| --- | --- | --- | --- | --- | --- | --- | --- | --- | --- | --- |
| (Hsiao et al., 1994) | 39/M | *Trichosporon* sp | Blood | Skin lesions, catheter tip, urine | NR^4^ | CONS^5^ | AML^6^ | Yes | Yes | Yes |
| (Nasu et al., 1994) | 60/M | *Trichosporon* sp | Blood | Lungs, liver, kidneys, thyroid, heart, skin | NR | NR | AML | Yes | Yes | Yes |
| (Higgins et al., 1994) | 1/M | *Trichosporon* sp | Skin | Gastric erosion | NR | NR | HL^7^ | Yes | NR | NR |
| (Hung et al., 1995) | 16/F | *Trichosporon* sp | Blood | NR | NR | NR | AML | Yes | Yes | NR |
| (Hung et al., 1995) | 40/F | *Trichosporon* sp | Blood | CVC tip | NR | NR | AML | No | Yes | Yes |
| (Grauer et al., 1994) | 50/M | *Trichosporon sp* | Blood | NR | NR | NR | AML | Yes | Yes | Yes |
| (Spánik et al., 1995) | 19/M | *Trichosporon* sp | Blood | NR | NR | *Klebsiella pneumoniae* | AML | Yes | Yes | Yes |
| (Hadley et al., 2002) | 78/NR | *Trichosporon* sp | Blood | NR | NR | NR | AML | Yes | No | Yes |
| (Hadley et al., 2002) | 78/NR | *Trichosporon* sp | Blood | NR | NR | NR | AML | No | No | Yes |
| (Itoh et al., 1996) | 5/F | *T. asahii* | Blood | Skin | NR | NR | ALL^8^ | Yes | Yes | Yes |
| (Fanci et al., 1997) | 63/M | *Trichosporon sp* | Blood | Lungs | NR | NR | MM^9^ | Yes | Yes | NR |
| (Kataoka-Nishimura et al., 1998) | 71/M | Trichosporon sp | Blood | NR | NR | NR | MDS^10^ | Yes | Yes | NR |
| (Kataoka-Nishimura et al., 1998) | 37/M | *Trichosporon sp* | Blood | Skin, Lungs | NR | NR | ALL, BMT^11^, GVHD^12^ | Yes | Yes | Yes |
| (Kataoka-Nishimura et al., 1998) | 50/F | *Trichosporon sp* | Blood | Skin, Lungs | NR | NR | AMxL^13^ | NR | Yes | Yes |
| (Kataoka-Nishimura et al., 1998) | 41/M | *Trichosporon sp* | Blood | Skin, Lungs | NR | NR | AML | Yes | Yes | Yes |
| (Kataoka-Nishimura et al., 1998) | 55/M | *Trichosporon sp* | Blood | Skin, Lungs | NR | NR | ALL | Yes | Yes | Yes |
| (Kataoka-Nishimura et al., 1998) | 66/M | *Trichosporon sp* | Blood | Skin, Lungs | NR | NR | HDD^14^ | Yes | Yes | Yes |
| (Kataoka-Nishimura et al., 1998) | 42/M | *Trichosporon sp* | Blood | Lungs | NR | NR | AML | Yes | Yes | NR |
| (Kataoka-Nishimura et al., 1998) | 66/M | *Trichosporon sp* | Blood | Lungs | NR | NR | AML | Yes | Yes | Yes |
| (Kataoka-Nishimura et al., 1998) | 36/M | *Trichosporon sp* | Blood | Lungs | NR | NR | ALL | Yes | Yes | Yes |
| (Sklair-Levy et al., 1998) | 51/M | *Trichosporon sp* | Blood | Lungs, spleen | NR | NR | AML | Yes | Yes | NR |
| (Krcmery et al., 1999) | NR/M | Trichosporon sp | Blood | NR | NR | No | AML | Yes | Yes | Yes |
| (Krcmery et al., 1999) | 35/M | Trichosporon sp | Blood | CVC tip | NR | NR | AML | Yes | Yes | Yes |
| (Krcmery et al., 1999) | 25/M | Trichosporon sp | Blood | NR | NR | NR | AML | Yes | Yes | NR |
| (Krcmery et al., 1999) | 24/F | Trichosporon sp | Blood | NR | NR | NR | AML | Yes | Yes | NR |
| (Krcmery et al., 1999) | 23/M | Trichosporon sp | Blood | NR | NR | NR | CLL^15^ | Yes | Yes | NR |
| (Krcmery et al., 1999) | 30/M | Trichosporon sp | Blood | NR | NR | NR | AML | Yes | Yes | NR |
| (Krcmery et al., 1999) | 49/F | Trichosporon sp | Blood | NR | NR | NR | AML | Yes | Yes | NR |
| (Krcmery et al., 1999) | 31/F | Trichosporon sp | Blood | NR | NR | NR | AML | Yes | Yes | NR |
| (Takamura et al., 1999) | 53/M | *T. asahii* | Skin | Blood | NR | NR | AML | Yes | Yes | Yes |
| (Erer et al., 2000) | 18/M | *Trichosporon sp* | Blood | NR | NR | NR | β-T^16^, BMT, GVHD | No | Yes | Yes |
| (Erer et al., 2000) | 17/M | *Trichosporon sp* | Blood | Skin | NR | NR | β-T, BMT, GVHD | Yes | Yes | NR |
| (Erer et al., 2000) | 8/F | *Trichosporon sp* | Blood | Skin | NR | NR | ALL, BCT^17^ | Yes | Yes | NR |
| (Moretti-Branchini et al., 2001) | 42/F | *T. asahii* | Blood | NR | NR | CMV^18^ | CML^19^ | Yes | Yes | NR |
| (Kim et al., 2001) | 41/M | *Trichosporon sp* | Blood | Skin, lungs | NR | NR | MDS | Yes | Yes | Yes |
| (Meyer et al., 2002) | 13/NR | *T. asahii* | Blood | Liver, spleen | NR | NR | BL | Yes | Yes | NR |
| (Fournier et al., 2002) | 45/M | *T. asahii* | Blood | Skin, liver, spleen, retina, lungs | Anti-GXM^20^ negative | NR | AML | Yes | Yes | Yes |
| (Marty et al., 2003) | 56/F | T. loubieri | Blood | liver, spleen | Anti-GXM negative | NR | ALL | Yes | Yes | NR |
| (Chang et al., 2003) | 44/M | Trichosporon sp | Blood | Skin, lungs | Anti-GXM negative | NR | MDS | Yes | Yes | NR |
| (Bassetti et al., 2004) | 54/M | *T. asahii* | Blood | NR | negative GM^21^ | NR | AML | Yes | Yes | Yes |
| (Chowdhary et al., 2004) | 41/M | *T.asahii* | Sputum | Blood | NR | NR | AML | Yes | Yes | Yes |
| (Viscomi et al., 2004) | 56/F | *Trichosporon sp* | Blood | Liver, spleen | NR | NR | ALL | NR | Yes | NR |
| (Antachopoulos et al., 2005) | 13/M | *T. asahii* | Blood | Skin, lungs | NR | NR | ALL | Yes | Yes | Yes |
| (Chan-Tack, 2005) | 43/M | *T. asahii* | Blood | CVC tip, skin, bone marrow, lungs | NR | NR | ALL | Yes | Yes | Yes |
| (Rodrigues et al., 2006) | 59/M | *T. asahii* | Blood | NR | NR | *Aspergillus flavus* | AML | Yes | Yes | Yes |
| (Matsue et al., 2006) | 56/M | *T. asahii* | Blood | BAL^22^ | NR | NR | AML | Yes | Yes | NR |
| (Matsue et al., 2006) | 55/M | *T. asahii* | Blood | NR | Positive GM | NR | AML | Yes | Yes | NR |
| (Matsue et al., 2006) | 60/M | *Trichosporon sp* | Blood | NR | NR | NR | MDS | NR | Yes | Yes |
| (Matsue et al., 2006) | 56/M | *Trichosporon sp* | Blood | Urine | NR | NR | AML | Yes | Yes | NR |
| (Kendirli et al., 2006) | 15/M | *T. mucoides/*  *dermatis* | Blood | NR | NR | NR | ALL | NR | Yes | NR |
| (Koyanagi et al., 2006) | 30/M | *T. inkin* | Blood | Lungs, kidneys,  liver, spleen, pancreas,  thyroid gland, skin, brain | NR | NR | AML | Yes | Yes | NR |
| (Akagi et al., 2006) | 72/M | *T.asahii* | Blood | NR | Positive β-D-Glucan | NR | AML | Yes | Yes | NR |
| (Akagi et al., 2006) | 79/F | *T. asahii* | Blood | NR | positive β-D-Glucan | NR | AML | yes | Yes | NR |
| (Ghiasian et al., 2006) | 11/F | *T.asahii* | Blood | Sputum, liver and lung biopsies | NR | NR | AA^23^, BMT | Yes | Yes | NR |
| (Meguro-Hashimoto et al., 2006) | 30/M | *Trichosporon* sp | Blood | Muscle | Negative GM, anti-GXM and b-d-glucan | NR | AML | Yes | NR | NR |
| (Miura et al., 2007) | 77M | *T. asahii* | Blood | Skin lesion | Positive β-D-Glucan | NR | CLL | Yes | Yes | NR |
| (Rieger et al., 2007) | 68/M | *T. asahii* | Blood | NR | NR | NR | AML | Yes | Yes | Yes |
| (Hara et al., 2007) | 64/M | *Trichosporon* sp | Blood | Eye | Positive β-D-Glucan | NR | AML | Yes | NR | NR |
| (Hara et al., 2007) | 43/F | *Trichosporon* sp | Blood | Eye | Positive β-D-Glucan | NR | ALL | Yes | NR | NR |
| (Hosoki et al., 2008) | 18/M | *T.asahii* | Blood | Catheter tip | Negative β-D-Glucan | NR | MDS, CBT | NR | Yes | Yes |
| (Bayramoglu et al., 2008) | 47/M | *T.asahii* | Blood | Lungs, skin | NR | NR | AML | Yes | Yes | NR |
| (Thibeault et al., 2008) | 11/M | *T.asahii* | Blood | Liver kidneys, spleen | NR | NR | ALL | Yes | Yes | Yes |
| (Tsuji et al., 2008) | 16/M | *T.asahii* | Urine | Blood | Positive anti-GXM and  B-D-Glucan | NR | ALL, BMT | Yes | Yes | NR |
| (Walia et al., 2009) | 78/M | *Trichosporon* sp | Blood | Skin lesion, eye | NR | NR | AML | Yes | NR | NR |
| (Fekkar et al., 2009) | NR | *T. dermatis* | Blood | BAL, skin and colic biopsies | Positive GM and  anti-GXM | NR | Aplasia, BCT | Yes | NR | NR |
| (Kudo et al., 2011) | 5m/F | *T. asahii* | Blood | NR | Positive anti-GXM and  B-D-glucan | NR | AML, CBT | Yes | Yes | NR |
| (Gabriel et al., 2011) | 21/F | *T. loubieri* | Blood | NR | NR | NR | T-LL^24^ | NR | Yes | Yes |
| (Menezes et al., 2012) | 31/M | *T. asahii* | Blood | NR | NR | NR | AML | Yes | NR | NR |
| (Hosokawa et al., 2012) | 21/M | *T.asahii* | Blood | NR | NR | NR | AML | Yes | Yes | No |
| (Odero et al., 2013) | 52/M | *T. asahii* | Blood | Skin lesions | NR | NR | ALL | NR | Yes | NR |
| (Chen et al., 2014) | NR | *T. asahii* | Blood | Blood from CVC | NR | NR | AML | Yes | Yes | Yes |
| (Karapinar et al., 2014) | 16/F | *T. asahii* | Blood | NR | NR | NR | AA | Yes | NR | NR |
| (Karapinar et al., 2014) | 5/F | *T. asahii* | Blood | NR | NR | NR | ALL | Yes | NR | NR |
| (Issarachaikul et al., 2014) | 38/M | *T. asahii* | CVC tip | Brain abscess | NR | NR | ALL | Yes | NR | Yes |
| (Tanyildiz et al., 2015) | 2/M | *T. asahii* | Blood | NR | NR | NR | LCH^36^ | Yes | Yes | Yes |
| (Tanyildiz et al., 2015) | 12/F | *T. asahii* | Blood | NR | NR | NR | CML | Yes | Yes | NR |
| (Capoor et al., 2015) | 19/F | *T. asahii* | Blood | NR | NR | NR | ALL | NR | NR | Yes |
| (Capoor et al., 2015) | 30/F | *T. japonicum* | Blood | NR | NR | NR | AML | NR | NR | NR |
| (Pérard et al., 2015) | 51/M | *T. faecale* | Blood | Skin lesions, oral cavity | NR | NR | AA | Yes | Yes | Yes |

Table S1 continued.

| Ref. | Breakthrough infection | Treatment | CVC  removal | Neutrophil recovery | Outcome |
| --- | --- | --- | --- | --- | --- |
| (Hsiao et al., 1994) | AMB^25^ | AMB | Yes | NR | Unfavorable |
| (Nasu et al., 1994) | NR | AMB | Yes | NR | Unfavorable |
| (Higgins et al., 1994) | AMB | FLU^26^ | NR | NR | Unfavorable |
| (Hung et al., 1995) | NR | No | NR | NR | Unfavorable |
| (Hung et al., 1995) | NR | AMB | Yes | NR | Unfavorable |
| (Grauer et al., 1994) | AMB | L-AMB^27^ | NR | NR | Unfavorable |
| (Spánik et al., 1995) | NR | AMB | Yes | Yes | Favorable |
| (Hadley et al., 2002) | NR | FLU | Yes | NR | Favorable |
| (Hadley et al., 2002) | NR | FLU | Yes | NA | Favorable |
| (Itoh et al., 1996) | NR | AMB | NR | Yes | Favorable |
| (Fanci et al., 1997) | NR | AMB | NR | Yes | Unfavorable |
| (Kataoka-Nishimura et al., 1998) | AMB | No | NR | NR | Unfavorable |
| (Kataoka-Nishimura et al., 1998) | AMB | AMB | NR | NR | Unfavorable |
| (Kataoka-Nishimura et al., 1998) | AMB | No | NR | NR | Unfavorable |
| (Kataoka-Nishimura et al., 1998) | AMB | AMB | NR | NR | Unfavorable |
| (Kataoka-Nishimura et al., 1998) | FLU | AMB | NR | NR | Unfavorable |
| (Kataoka-Nishimura et al., 1998) | ITRA^28^ | AMB | NR | NR | Unfavorable |
| (Kataoka-Nishimura et al., 1998) | NR | AMB | NR | Yes | Unfavorable |
| (Kataoka-Nishimura et al., 1998) | AMB | MIC^9^ | NR | NR | Unfavorable |
| (Kataoka-Nishimura et al., 1998) | AMB | AMB | NR | NR | Unfavorable |
| (Sklair-Levy et al., 1998) | AMB | ITRA | NR | NR | Favorable |
| (Krcmery et al., 1999) | ITRA | AMB+FLU | Yes | NR | Favorable |
| (Krcmery et al., 1999) | ITRA | AMB+FLU | Yes | NR | Favorable |
| (Krcmery et al., 1999) | AMB | AMB | NR | NR | Unfavorable |
| (Krcmery et al., 1999) | NR | AMB | NR | NR | Unfavorable |
| (Krcmery et al., 1999) | NR | AMB | NR | NR | Unfavorable |
| (Krcmery et al., 1999) | NR | AMB | NR | NR | Unfavorable |
| (Krcmery et al., 1999) | AMB | AMB | NR | NR | Unfavorable |
| (Krcmery et al., 1999) | NR | AMB | NR | NR | Unfavorable |
| (Takamura et al., 1999) | FLU | AMB | NR | NR | Unfavorable |
| (Erer et al., 2000) | AMB | DC-AMB^29^  +FLU | Yes | Yes | Favorable |
| (Erer et al., 2000) | AMB | AMB | NR | Yes | Unfavorable |
| (Erer et al., 2000) | AMB | FLU | NR | NR | Unfavorable |
| (Moretti-Branchini et al., 2001) | ITRA | AMB | NR | NR | Unfavorable |
| (Kim et al., 2001) | AMB | AMB+  ITRA | Yes | Yes | Favorable |
| (Meyer et al., 2002) | AMB | ITRA | NR | Yes | Favorable |
| (Fournier et al., 2002) | AMB+  FLU | VOR^30^ | Yes | Yes | Favorable |
| (Marty et al., 2003) | AMB+  5-FC^31^, LC-AMB | ITRA | NR | Yes | Favorable |
| (Chang et al., 2003) | AMB | AMB+  FLU | NR | NR | Favorable |
| (Bassetti et al., 2004) | FLU, VOR | L-AMB+  CAS^32^ | NR | NR | Favorable |
| (Chowdhary et al., 2004) | NR | AMB | Yes | NR | Unfavorable |
| (Viscomi et al., 2004) | L-AMB | L-AMB+  FLU | NR | NR | Unfavorable |
| (Antachopoulos et al., 2005) | L-AMB | VOR | NR | NR | Favorable |
| (Chan-Tack, 2005) | AMB | VOR | Yes | NR | Unfavorable |
| (Rodrigues et al., 2006) | NR | FLU, AMB, ITRA,  L-AMB+  CAS | NR | NR | Unfavorable |
| (Matsue et al., 2006) | MIC+  AMB | FLU | NR | NR | Unfavorable |
| (Matsue et al., 2006) | MIC | VOR | NA | Yes | Favorable |
| (Matsue et al., 2006) | MIC | FLU | Yes | NR | Favorable |
| (Matsue et al., 2006) | MIC | FLU | NR | NR | Unfavorable |
| (Kendirli et al., 2006) | NR | LC-AMB^33^+  5-FC | NR | Yes | Favorable |
| (Koyanagi et al., 2006) | MIC+  AMB | MIC+  AMB | NR | NR | Unfavorable |
| (Akagi et al., 2006) | MIC | AMB+  MCZ^34^ | NR | No | Unfavorable |
| (Akagi et al., 2006) | MIC | FLU | NR | Yes | Favorable |
| (Ghiasian et al., 2006) | AMB | AMB | NR | NR | Unfavorable |
| (Meguro-Hashimoto et al., 2006) | FLU | AMB | NR | NR | Favorable |
| (Miura et al., 2007) | ITRA | No | NR | NR | Unfavorable |
| (Rieger et al., 2007) | POS | L-AMB  +VOR | Yes | Yes | Favorable |
| (Hara et al., 2007) | FLU | AMB | NR | NR | Favorable |
| (Hara et al., 2007) | FLU, VOR | AMB | NR | NR | Favorable |
| (Hosoki et al., 2008) | VOR | L-AMB  +ITRA | Yes | NR | Favorable |
| (Bayramoglu et al., 2008) | CAS | L-AMB  +VOR | NR | NR | Unfavorable |
| (Thibeault et al., 2008) | L-AMB | VOR | Yes | NR | Unfavorable |
| (Tsuji et al., 2008) | MIC | VOR | NR | Yes | Favorable |
| (Walia et al., 2009) | MIC | MIC+VOR | NR | Yes | Favorable |
| (Fekkar et al., 2009) | CAS | VOR | NR | NR | Unfavorable |
| (Kudo et al., 2011) | FLU | VOR | NR | Yes | Favorable |
| (Gabriel et al., 2011) | No | VOR | Yes | NR | Favorable |
| (Menezes et al., 2012) | NR | AMB+CAS | NR | NR | Favorable |
| (Hosokawa et al., 2012) | MIC | L-AMB+  VOR | NR | NR | Favorable |
| (Odero et al., 2013) | No | AMB | NR | NR | Favorable |
| (Chen et al., 2014) | VOR | L-AMB+  CAS | NR | Yes | Favorable |
| (Karapinar et al., 2014) | CAS | VOR | NR | NR | Unfavorable |
| (Karapinar et al., 2014) | CAS | VOR | NR | Yes | Favorable |
| (Issarachaikul et al., 2014) | AMB, CAS | VOR | Yes | NR | Favorable |
| (Tanyildiz et al., 2015) | L-AMB | L-AMB+VOR | Yes | Yes | Favorable |
| (Tanyildiz et al., 2015) | NR | L-AMB+VOR | NR | Yes | Favorable |
| (Capoor et al., 2015) | FLU | No | NR | NR | Unfavorable |
| (Capoor et al., 2015) | FLU | AMB | NR | NR | Unfavorable |
| (Pérard et al., 2015) | CAS | L-AMB+VOR | Yes | Yes | Favorable |

^1^ Neutropenia: below 500 cells/mm^3^; ^2^ATB: previous antibiotic therapy; ^3^CVC: central venous catheter; ^4^NR: not reported; ^5^CONS: coagulase-negative *Staphylococcus* species; ^6^AML: acute myeloid leukemia; ^7^HL: haemophagocytic lymphohistiocytosis; ^8^ALL: acute lymphoid leukemia; ^9^ MM: multiple myeloma; ^10^ MDS: myelodisplastic syndrome; ^11^ BMT: bone marrow transplant; ^12^GVHD: graft versus host disease;  ^13^AMxL: acute mixed leukemia; ^14^HD: Hodgkin’s disease; ^15^CLL: chronic lymphoid leukemia; ^16^β-T: β-thalassemia; ^17^ BCT: blood cord transplant; ^18^ CMV: cytomegalovirus; ^19^ CML: chronic myeloid leukemia;  ^20^ anti-GXM: cryptococcal antigen detection assay (glucuronoxylomannan); ^21^ GM: galactomannan detection assay; ^22^BAL: bronchoalveolar lavage; ^23^AA: aplastic anemia; ^24^T-LL: T-lymphoblastic lymphoma; ^25^AMB: amphotericin B deoxicolate; ^26^FLU: fluconazole; ^27^L-AMB: liposomal amphotericin B; ^28^ ITRA: itraconazole;  ^29^ MIC: Micafungin; ^30^ CD-AMB: colloidal dispersion amphotericin B; ^31^ VOR: voriconazole;  ^32^5-FC: 5-fluorocytosin; ^33^CAS: caspofungin; ^34^ LC-AMB: lipid complex amphotericin B; ^35^MCZ: miconazole, ^36^ LCH: Langerhans cell histiocytosis.

**References**

Akagi, T., Yamaguti, K., Kawamura, T., Nakumura, T., Kubo, K., and Takemori, H. (2006). Breakthrough trichosporonosis in patients with acute myeloid leukemia receiving micafungin. *Leuk. Lymphoma* 47, 1182–1183. doi:10.1080/10428190500272499.

Antachopoulos, C., Papakonstantinou, E., Dotis, J., Bibashi, E., Tamiolaki, M., Koliouskas, D., et al. (2005). Fungemia due to Trichosporon asahii in a neutropenic child refractory to amphotericin B: clearance with voriconazole. *J. Pediatr. Hematol. Oncol.* 27, 283–285.

Bassetti, M., Bisio, F., Di Biagio, A., Pierri, I., Balocco, M., Soro, O., et al. (2004). Trichosporon asahii infection treated with caspofungin combined with liposomal amphotericin B. *J. Antimicrob. Chemother.* 54, 575–577. doi:10.1093/jac/dkh337.

Bayramoglu, G., Sonmez, M., Tosun, I., Aydin, K., and Aydin, F. (2008). Breakthrough Trichosporon asahii fungemia in neutropenic patient with acute leukemia while receiving caspofungin. *Infection* 36, 68–70. doi:10.1007/s15010-007-6278-6.

Capoor, M. R., Gupta, D. K., Verma, P. K., and Sachdeva, H. C. (2015). Rare yeasts causing fungemia in immunocompromised and haematology patients: Case series from Delhi. *Indian J. Med. Microbiol.* 33, 576–579. doi:10.4103/0255-0857.167320.

Chang, S. E., Kim, K. J., Lee, W. S., Choi, J. H., Sung, K. J., Moon, K. C., et al. (2003). A case of Trichosporon cutaneum folliculitis and septicaemia. *Clin. Exp. Dermatol.* 28, 37–38.

Chan-Tack, K. M. (2005). Fatal Trichosporon asahii septicemia in a Guatemalan farmer with acute lymphoblastic leukemia. *South. Med. J.* 98, 954–955. doi:10.1097/01.smj.0000177350.91609.ea.

Chen, J., Chen, F., Wang, Y., Yang, L.-Y., Miao, M., Han, Y., et al. (2014). Use of combination therapy to successfully treat breakthrough Trichosporon asahii infection in an acute leukemia patient receiving voriconazole. *Med. Mycol. Case Rep.* 6, 55–57. doi:10.1016/j.mmcr.2014.09.003.

Chowdhary, A., Ahmad, S., Khan, Z. U., Doval, D. C., and Randhawa, H. S. (2004). Trichosporon asahii as an emerging etiologic agent of disseminated trichosporonosis: a case report and an update. *Indian J. Med. Microbiol.* 22, 16–22.

Erer, B., Galimberti, M., Lucarelli, G., Giardini, C., Polchi, P., Baronciani, D., et al. (2000). Trichosporon beigelii: a life-threatening pathogen in immunocompromised hosts. *Bone Marrow Transplant.* 25, 745–749. doi:10.1038/sj.bmt.1702231.

Fanci, R., Pecile, P., Martinez, R. L., Fabbri, A., and Nicoletti, P. (1997). Amphotericin B treatment of fungemia due to unusual pathogens in neutropenic patients: report of two cases. *J. Chemother. Florence Italy* 9, 427–430. doi:10.1179/joc.1997.9.6.427.

Fekkar, A., Brun, S., D’Ussel, M., Uzunov, M., Cracco, C., Dhédin, N., et al. (2009). Serum cross-reactivity with Aspergillus galactomannan and cryptococcal antigen during fatal disseminated Trichosporon dermatis infection. *Clin. Infect. Dis. Off. Publ. Infect. Dis. Soc. Am.* 49, 1457–1458. doi:10.1086/644499.

Fournier, S., Pavageau, W., Feuillhade, M., Deplus, S., Zagdanski, A.-M., Verola, O., et al. (2002). Use of voriconazole to successfully treat disseminated Trichosporon asahii infection in a patient with acute myeloid leukaemia. *Eur. J. Clin. Microbiol. Infect. Dis. Off. Publ. Eur. Soc. Clin. Microbiol.* 21, 892–896. doi:10.1007/s10096-002-0841-y.

Gabriel, F., Noel, T., and Accoceberry, I. (2011). Fatal invasive trichosporonosis due to Trichosporon loubieri in a patient with T-lymphoblastic lymphoma. *Med. Mycol.* 49, 306–310. doi:10.3109/13693786.2010.525758.

Ghiasian, S. A., Maghsood, A. H., and Mirhendi, S. H. (2006). Disseminated, fatal Trichosporon asahii infection in a bone marrow transplant recipient. *J. Microbiol. Immunol. Infect. Wei Mian Yu Gan Ran Za Zhi* 39, 426–429.

Grauer, M. E., Bokemeyer, C., Bautsch, W., Freund, M., and Link, H. (1994). Successful treatment of a Trichosporon beigelii septicemia in a granulocytopenic patient with amphotericin B and granulocyte colony-stimulating factor. *Infection* 22, 283–286.

Hadley, S., Martinez, J. A., McDermott, L., Rapino, B., and Snydman, D. R. (2002). Real-time antifungal susceptibility screening aids management of invasive yeast infections in immunocompromised patients. *J. Antimicrob. Chemother.* 49, 415–419.

Hara, S., Yokote, T., Oka, S., Akioka, T., Kobayashi, K., Hirata, Y., et al. (2007). Endophthalmitis due to Trichosporon beigelii in acute leukemia. *Int. J. Hematol.* 85, 415–417. doi:10.1532/IJH97.06228.

Higgins, E. M., Layton, D. M., Arya, R., Salisbury, J., and du Vivier, A. W. (1994). Disseminated Trichosporon beigelii infection in an immunosuppressed child. *J. R. Soc. Med.* 87, 292–293.

Hosokawa, K., Yamazaki, H., Mochizuki, K., Ohata, K., Ishiyama, K., Hayashi, T., et al. (2012). Successful treatment of Trichosporon fungemia in a patient with refractory acute myeloid leukemia using voriconazole combined with liposomal amphotericin B. *Transpl. Infect. Dis. Off. J. Transplant. Soc.* 14, 184–187. doi:10.1111/j.1399-3062.2011.00670.x.

Hosoki, K., Iwamoto, S., Kumamoto, T., Azuma, E., and Komada, Y. (2008). Early detection of breakthrough trichosporonosis by serum PCR in a cord blood transplant recipient being prophylactically treated with voriconazole. *J. Pediatr. Hematol. Oncol.* 30, 917–919. doi:10.1097/MPH.0b013e3181864aa7.

Hsiao, G. H., Chang, C. C., Chen, J. C., Kuo, W. L., and Huang, S. F. (1994). Trichosporon beigelii fungemia with cutaneous dissemination. A case report and literature review. *Acta Derm. Venereol.* 74, 481–482.

Hung, C. C., Chang, S. C., Chen, Y. C., Tien, H. F., and Hsieh, W. C. (1995). Trichosporon beigelii fungemia in patients with acute leukemia: report of three cases. *J. Formos. Med. Assoc. Taiwan Yi Zhi* 94, 127–131.

Issarachaikul, R., Lertwannawit, O., Detporntewan, P., Uaprasert, N., Plongla, R., Shuangshoti, S., et al. (2014). Catheter-related bloodstream infections caused by Trichosporon species. *Southeast Asian J. Trop. Med. Public Health* 45, 421–429.

Itoh, T., Hosokawa, H., Kohdera, U., Toyazaki, N., and Asada, Y. (1996). Disseminated infection with Trichosporon asahii. *Mycoses* 39, 195–199.

Karapinar, D. Y., Karadaş, N., Yazici, P., Polat, S. H., and Karapinar, B. (2014). Trichosporon asahii, sepsis, and secondary hemophagocytic lymphohistiocytosis in children with hematologic malignancy. *Pediatr. Hematol. Oncol.* 31, 282–284. doi:10.3109/08880018.2013.851754.

Kataoka-Nishimura, S., Akiyama, H., Saku, K., Kashiwa, M., Mori, S., Tanikawa, S., et al. (1998). Invasive infection due to Trichosporon cutaneum in patients with hematologic malignancies. *Cancer* 82, 484–487.

Kendirli, T., Ciftçi, E., Ince, E., Oncel, S., Dalgiç, N., Güriz, H., et al. (2006). Successful treatment of Trichosporon mucoides infection with lipid complex amphotericin B and 5-fluorocytosine. *Mycoses* 49, 251–253. doi:10.1111/j.1439-0507.2006.01223.x.

Kim, J. C., Kim, Y. S., Park, C. S., Kang, J. M., Kim, B. N., Woo, J. H., et al. (2001). A case of disseminated Trichosporon beigelii infection in a patient with myelodysplastic syndrome after chemotherapy. *J. Korean Med. Sci.* 16, 505–508.

Koyanagi, T., Nishida, N., Osabe, S., Imamura, Y., Yamamoto, S., Shichiji, A., et al. (2006). Autopsy case of disseminated Trichosporon inkin infection identified with molecular biological and biochemical methods. *Pathol. Int.* 56, 738–743. doi:10.1111/j.1440-1827.2006.02040.x.

Krcmery, V., Mateicka, F., Kunová, A., Spánik, S., Gyarfás, J., Sycová, Z., et al. (1999). Hematogenous trichosporonosis in cancer patients: report of 12 cases including 5 during prophylaxis with itraconazol. *Support. Care Cancer Off. J. Multinatl. Assoc. Support. Care Cancer* 7, 39–43.

Kudo, K., Terui, K., Sasaki, S., Kamio, T., Sato, T., and Ito, E. (2011). Voriconazole for both successful treatment of disseminated Trichosporon asahii infection and subsequent cord blood transplantation in an infant with acute myelogenous leukemia. *Bone Marrow Transplant.* 46, 310–311. doi:10.1038/bmt.2010.96.

Marty, F. M., Barouch, D. H., Coakley, E. P., and Baden, L. R. (2003). Disseminated trichosporonosis caused by Trichosporon loubieri. *J. Clin. Microbiol.* 41, 5317–5320.

Matsue, K., Uryu, H., Koseki, M., Asada, N., and Takeuchi, M. (2006). Breakthrough trichosporonosis in patients with hematologic malignancies receiving micafungin. *Clin. Infect. Dis. Off. Publ. Infect. Dis. Soc. Am.* 42, 753–757. doi:10.1086/500323.

Meguro-Hashimoto, A., Takatoku, M., Ohmine, K., Toshima, M., Mori, M., Nagai, T., et al. (2006). The usefulness of magnetic resonance imaging (MRI) for disseminated trichosporosis of the gastrocnemius muscles. *J. Infect.* 53, e135–138. doi:10.1016/j.jinf.2005.11.026.

Menezes, E. A., Marinho, J. A. de S., Angelo, M. R. F., Cunha, M. da C. dos S. O., Cunha, F. A., and Vasconcelos Júnior, A. A. de (2012). Isolation and antifungal susceptibility testing of Trichosporon asahii in Ceará, Brazil. *Rev. Inst. Med. Trop. São Paulo* 54, 1–3.

Meyer, M. H., Letscher-Bru, V., Waller, J., Lutz, P., Marcellin, L., and Herbrecht, R. (2002). Chronic disseminated Trichosporon asahii infection in a leukemic child. *Clin. Infect. Dis. Off. Publ. Infect. Dis. Soc. Am.* 35, e22–25. doi:10.1086/340983.

Miura, Y., Kaneko, M., Nishizawa, M., Okamoto, K., Hirai, M., Kaneko, H., et al. (2007). Breakthrough infection of Trichosporon asahii in a patient with chronic lymphocytic leukemia. *Int. J. Hematol.* 85, 177–178. doi:10.1532/IJH97.06220.

Moretti-Branchini, M. L., Fukushima, K., Schreiber, A. Z., Nishimura, K., Papaiordanou, P. M., Trabasso, P., et al. (2001). Trichosporon species infection in bone marrow transplanted patients. *Diagn. Microbiol. Infect. Dis.* 39, 161–164.

Nasu, K., Akizuki, S., Yoshiyama, K., Kikuchi, H., Higuchi, Y., and Yamamoto, S. (1994). Disseminated Trichosporon infection. A case report and immunohistochemical study. *Arch. Pathol. Lab. Med.* 118, 191–194.

Odero, V., Galán-Sánchez, F., García-Agudo, L., García-Tapia, A. M., Guerrero-Lozano, I., and Rodríguez-Iglesias, M. A. (2013). [Fungemia due to Trichosporon asahii in a patient with hematological malignancy.]. *Rev. Iberoam. Micol.* doi:10.1016/j.riam.2013.09.001.

Pérard, B., Rougeron, A., Favre, S., Accoceberry, I., Vigouroux, S., Mohr, C., et al. (2015). Trichosporon faecale invasive infection in a patient with severe aplastic anemia: Efficacy of voriconazole and liposomal amphotericin B before neutrophil recovery. *Med. Mycol. Case Rep.* 9, 12–14. doi:10.1016/j.mmcr.2015.06.003.

Rieger, C., Geiger, S., Herold, T., Nickenig, C., and Ostermann, H. (2007). Breakthrough infection of Trichosporon asahii during posaconazole treatment in a patient with acute myeloid leukaemia. *Eur. J. Clin. Microbiol. Infect. Dis. Off. Publ. Eur. Soc. Clin. Microbiol.* 26, 843–845. doi:10.1007/s10096-007-0366-5.

Rodrigues, G. da S., de Faria, R. R. U., Guazzelli, L. S., Oliveira, F. de M., and Severo, L. C. (2006). [Nosocomial infection due to Trichosporon asahii: clinical revision of 22 cases]. *Rev. Iberoam. Micol.* 23, 85–89.

Sklair-Levy, M., Libson, Y., Lossos, I. S., and Bugomolsky-Yahalom, V. (1998). Splenic calcifications caused by Trichosporon beigelli infection: CT and ultrasound demonstration. *Eur. Radiol.* 8, 922–924. doi:10.1007/s003300050488.

Spánik, S., Kollár, T., Gyarfás, J., Kunová, A., and Krcméry, V. (1995). Successful treatment of catheter-associated fungemia due to Candida krusei and Trichosporon beigelii in a leukemic patient receiving prophylactic itraconazole. *Eur. J. Clin. Microbiol. Infect. Dis. Off. Publ. Eur. Soc. Clin. Microbiol.* 14, 148–149.

Takamura, S., Oono, T., Kanzaki, H., and Arata, J. (1999). Disseminated Trichosporonosis with Trichosporon asahii. *Eur. J. Dermatol. EJD* 9, 577–579.

Tanyildiz, H. G., Yesil, S., Toprak, S., Candir, M. O., and Sahin, G. (2015). Two Case Presentations Infected by Trichosporon asahii and Treated with Voriconazole Successfully. *Case Rep. Infect. Dis.* 2015, 651315. doi:10.1155/2015/651315.

Thibeault, R., Champagne, M., de Repentigny, L., Fournet, J.-C., Tapiero, B., Moghrabi, A., et al. (2008). Fatal disseminated Trichosporon asahii infection in a child with acute lymphoblastic leukemia. *Can. J. Infect. Dis. Med. Microbiol. J. Can. Mal. Infect. Microbiol. Médicale AMMI Can.* 19, 203–205.

Tsuji, Y., Tokimatsu, I., Sugita, T., Nozaki, M., Kobayashi, D., Imai, K., et al. (2008). Quantitative PCR assay used to monitor serum Trichosporon asahii DNA concentrations in disseminated trichosporonosis. *Pediatr. Infect. Dis. J.* 27, 1035–1037. doi:10.1097/INF.0b013e318179260d.

Viscomi, S. G., Mortelé, K. J., Cantisani, V., Glickman, J., and Silverman, S. G. (2004). Fatal, complete splenic infarction and hepatic infection due to disseminated Trichosporon beigelii infection: CT findings. *Abdom. Imaging* 29, 228–230. doi:10.1007/s00261-003-0099-6.

Walia, H., Tucci, V. T., Greene, J. N., Tordilla-Wadia, J., Kelty, P., and Walia, S. (2009). A case of endogenous trichosporon endophthalmitis treated with micafungin and voriconazole. *J. Glob. Infect. Dis.* 1, 71–74. doi:10.4103/0974-777X.52987.
