# Supplementary material for: Invasive Trichosporon Infection: a Systematic Review on a Re-emerging Fungal Pathogen
Source: Front Microbiol. 2016 Oct 17;7:1629. doi: 10.3389/fmicb.2016.01629 (PMC5065970; doi:10.3389/fmicb.2016.01629)
Supplement: Supplementary file 2 [file Table2.DOCX]

**Table S2**. Summary of the forty-one cases of invasive trichosporonosis in patients with other immune disorders.

| Ref. | Type of  Infection | Age/  Sex | Species | First  isolate | Other  sites | Serological  tests | Other  pathogens | Baseline  disease |
| --- | --- | --- | --- | --- | --- | --- | --- | --- |
| (Lascaux et al., 1998) | Disseminated | 36/M | *Trichosporon* spp. | CSF^1^ | Skin | NR^2^ | NR | HIV, T-CD4+  9 cells/ml |
| (Ebright et al., 2001) | Disseminated | 53/F | *T.asahii* | Blood | Lung, heart,  kidneys, spleen,  brain | NR | NR | Diabetes,  pneumonia,  ARF^3^,  toxic epidermal  necrolysis,  corticosteroids |
| (Abliz et al., 2002) | Disseminated | 20/  F | *T.asahii* | Skin and  liver | NR | NR | NR | Cutaneus lupus,  corticosteroids |
| (Nettles et al., 2003) | Disseminated | 64/M | *T. mucoides/*  *dermatis* | Skin  biopsy | NR | NR | NR | Heart and  Kidney  transplant  recipient,  corticosteroids,  tacrolimus |
| (Abdala et al., 2005) | Disseminated | 54/F | *T.asahii* | Skin biopsy | Urine, BAL^4^ | NR | NR | Liver transplant |
| (Karabay et al., 2006) | Disseminated | 75/F | *T.asahii* | Blood | NR | NR | NR | Bladder Cancer,  ARF,  cystectomy and  ileal conduit  surgery |
| (Rodrigues et al., 2006) | Disseminated | 72/M | *T.asahii* | Blood | Urine | NR | NR | COPD^5^,  lung transplant  recipient,  corticosteroids |
| (Rodrigues et al., 2006) | Disseminated | 1/F | *T.asahii* | Blood | NR | NR | NR | Neuroblastoma,  neutropenia,  chemotherapy |
| (Gunn et al., 2006) | Disseminated | 1/M | *T. dermatis* | Blood | NR | NR | CONS^6^ | Autoimmune  enteropathy,  corticosteroids,  tacrolimus |
| (Gross and Kan, 2008) | Disseminated | 49/M | *T.asahii* | Blood | Urine | anti-GXM^7^  positive | NR | AIDS, diabetes, hepatitis C,  iv drug user,  TCD4+ 5 cells/ml |
| (David et al., 2008) | Disseminated | 28/M | *T. inkin* | Blood | Skin | negative  anti-GXM | Histoplasma  capsulatum | HIV, CD4+ 2/mm^3^ |
| (Chagas-Neto et al., 2009) | Disseminated | 49/M | *T.asahii* | Blood | NR | NR | NR | Lung neoplasia |
| (Chagas-Neto et al., 2009) | Disseminated | 33/M | *T.asahii* | Blood | NR | NR | NR | Kidney  transplant |
| (Lacasse and Cleveland, 2009) | Disseminated | 51/M | *T. mucoides/*  *dermatis* | Blood | NR | negative  GM^8^  and  anti-GXM | CONS | Liver tranplant  recipient,  tacrolimus |
| (Servonnet et al., 2010) | Disseminated | 77/M | *T.asahii* | Blood | NR | NR | NR | Rectal and  prostate  adenocarcinome,  ARF |
| (Tsai et al., 2012) | Disseminated | 54/NR | *T.asahii* | Blood | NR | NR | NR | Breast Cancer |
| (Tsai et al., 2012) | Disseminated | 61/NR | *T.asahii* | Blood | NR | NR | NR | Lung Cancer |
| (Tsai et al., 2012) | Disseminated | 61/NR | *T.asahii* | Blood | NR | NR | NR | Lung Cancer |
| (Tsai et al., 2012) | Disseminated | 41/NR | *T.asahii* | Blood | NR | NR | NR | CRF^9^, Kidney  transplant  recipient,  tuberculosis |
| (Hirschi et al., 2012) | Disseminated | 35/M | *T. mycotoxinovorans* | Blood | Thoracic  wound  abscess,  heart | positive GM | *Aspergillus*  *fumigatus*  *Scedosporium*  *apiospermum* | Cystic fibrosis,  lung transplant  recipient,  corticosteroids,  tacrolimus,  mycofenolate  mofetil and  basiliximab |
|  | Disseminated | 61/NR | *T.asahii* | Blood | NR | NR | NR | Lung Cancer |
| (Ozkaya-Parlakay et al., 2013) | Disseminated | 16/M | *T. asahii* | Blood | Sinus | NR | Mucorales | Ewing Sarcoma,  chemotherapy |
| (Yang et al., 2014) | Disseminated | 67/M | *T. asahii* | Blood | Sputum | NR | NR | Kidney  transplant,  diabetes, ARF,  corticosteroids,  tacrolimus,  mycofenolate  mofetil |
| (Nobrega de Almeida Júnior et al., 2015) | Disseminated | 71/M | *T. inkin* | Blood | NR | NR | NR | Pemphigus foliaceus, diabetes, ARF |
| (Nobrega de Almeida Júnior et al., 2015) | Disseminated | 9/F | *T. inkin* | Blood | NR | NR | NR | Pemphigus vulgaris, corticosteroids |
| (Chaitanya et al., 2015) | Disseminated | 45/M | *Trichosporon spp.* | Skin, muscle, lung | NR | NR | NR | Kidney transplant |
| (Piwoz et al., 2000) | Lung abscess | 9/M | *T. inkin* | Chest  wall  and lung | Lung | NR | NR | Chronic  granulomatous  disease |
| (Wynne et al., 2004) | Lung abscess | 9/F | *T. inkin* | Lung | NR | NR | NR | Chronic  granulomatous  disease |
| (Wynne et al., 2004) | Lung abscess | 13/M | *T. inkin* | Lung | NR | NR | NR | Chronic  granulomatous  disease |
| (Canales et al., 1998) | Pneumonia | 42/F | *Trichosporon* spp. | Sputum | NR | NR | NR | Breast  carcinoma,  neutropenia,  chemotherapy |
| (Songcharoen et al., 2011) | Pneumonia | 48/M | *T.asahii* | Lung | NR | Positive  assay with  monocolonal  anti-body | No | Rheumathoid  arthritis  mixed  connective tissue  disease,  adalimumab,  corticosteroids,  methotrexate |
| (Almeida Júnior et al., 2014) | Pneumonia | 42/M | *T. inkin* | Pleural  empiema | NR | NR | No | Idiopathic  dilated  cardiomyopathy,  corticosteroids,  heart transplant  recipient |
| [22] | Mediastinitis | 19/M | *T. inkin* | CM^10^ | NR | NR | No | Cystic fibrosis,  lung transplant,  corticosteroids,  tacrolimus,  azathioprine |
| (Kahana et al., 2003) | Intra-abdominal infection | 8/F | *Trichosporon* spp. | GB^11^ | NR | NR | NR | Natural Killer  Cell Deficiency |
| (Biasoli et al., 2008) | Intra-abdominal  infection | 23/F | *T. asahii* | Intra-cavitary  abscess | NR | NR | NR | Liver transplant  recipient |
| (Fadhil et al., 2011) | Intra-abdominal infection | 40/M | *Trichosporon* | Peri-  renal  abscess | renal  artery | NR | NR | Kidney  transplant  recipient |
| (Anuradha et al., 2000) | Peritonitis | 25/M | *Trichosporon* | PF^12^ | NR | NR | NR | HIV, T-CD4+  338 cells/ml |
| (Chen et al., 2013) | Peritonitis | 39/F | *T. mucoides/*  *dermatis* | PF | NR | NR | *Enterococcus* | Systemic lupus  erythematosus,  CRF |
| (Macêdo et al., 2011) | Esophagitis | 54/F | *T. inkin* | Eso-  phagus  biopsy | NR | NR | NR | Lung Cancer,  neutropenia,  chemotherapy |
| (Chakrabarti et al., 2002) | generalyzed  lymphadenopathy | 10/M | *T. asahii* | Axilary  and  cervical  lymph-  nodes | NR | anti-GXM  negative | NR | Job's Syndrome |
| (Basiri et al., 2012) | Brain abscess | 34/M | *T. asahii* | Brain  abscess | No | NR | NR | Autoimmune  hepatitis,  corticosteroids |
| (Ogura et al., 2012) | Granulomatous  interstitial  nephritis | 73/M | *T. laibachii* | Kidney  biopsy | NR | beta-D-glucan positive | Candida  albicans  catheter | Pharingeal  cancer,  chemotherapy |
|  |  |  |  |  |  |  |  |  |

Table S2 continued.

| Ref. | Type of Infection | AB^13^ | Invasive disposal | BRT^14^ | Treatment | Invasive disposal  removal | Outcome |
| --- | --- | --- | --- | --- | --- | --- | --- |
| (Lascaux et al., 1998))1 | Disseminated | NR | NR | NR | AMB^15^ | NR | Favorable |
| (Ebright et al., 2001))2 | Disseminated | Yes | CVC^16^ | NR | AMB+FLU^17^ | NR | Unfavorable |
| (Abliz et al., 2002))3 | Disseminated | Yes | NR | NR | AMB+FLU | NA | Favorable |
| (Nettles et al., 2003))4 | Disseminated | Yes | NR | NR | FLU | NA | Favorable |
| (Abdala et al., 2005))5 | Disseminated | NR | NR | NR | AMB | NA | Unfavorable |
| (Karabay et al., 2006))6 | Disseminated | Yes | CVC | FLU | CAS^18^ | NR | Unfavorable |
| (Rodrigues et al., 2006))7 | Disseminated | Yes | CVC | NR | FLU | NR | Unfavorable |
| (Rodrigues et al., 2006))7 | Disseminated | Yes | CVC | NR | AMB | NR | Favorable |
| (Gunn et al., 2006))8 | Disseminated | Yes | CVC | NR | VOR^19^ | Yes | Favorable |
| (Gross and Kan, 2008))9 | Disseminated | Yes | CVC | AMB | AMB+FLU | Yes | Unfavorable |
| (David et al., 2008)10 | Disseminated | NR | NR | AMB | VOR | NR | Favorable |
| (Chagas-Neto et al., 2009)11 | Disseminated | NR | CVC | NR | No | NR | Unfavorable |
| (Chagas-Neto et al., 2009)11 | Disseminated | NR | CVC | NR | FLU | NR | Unfavorable |
| (Lacasse and Cleveland, 2009)12 | Disseminated | Yes | NR | AND | POS^20^ | NR | Favorable |
| (Servonnet et al., 2010)13 | Disseminated | NR | NR | CAS | FLU | NR | Favorable |
| (Tsai et al., 2012)14 | Disseminated | NR | CVC | NR | FLU | NR | Favorable |
| (Tsai et al., 2012)14 | Disseminated | NR | CVC | NR | FLU | NR | Favorable |
| (Tsai et al., 2012)14 | Disseminated | NR | CVC | NR | FLU | NR | Favorable |
| (Tsai et al., 2012)14 | Disseminated | NR | CVC | NR | FLU | NR | Favorable |
| (Hirschi et al., 2012)15 | Disseminated | Yes | NR | CAS | CAS+VOR | NR | Unfavorable |
| (Ozkaya-Parlakay et al., 2013)16 | Disseminated | Yes | CVC | NR | CAS | NR | Unfavorable |
| (Yang et al., 2014)17 | Disseminated | Yes | NR | NR | CAS | NR | Unfavorable |
| ^18^(Nobrega de Almeida Júnior et al., 2015) | Disseminated | Yes | CVC | NR | L-AMB | NR | Unfavorable |
| ^18^(Nobrega de Almeida Júnior et al., 2015) | Disseminated | Yes | CVC | NR | FLU, VOR | NR | Favorable |
| (Chaitanya et al., 2015)19 | Disseminated | Yes | NR | NR | VOR | NR | Favorable |
| (Piwoz et al., 2000)20 | Lung abscess | Yes | NR | ITRA | AMB+FLU | NA | Favorable |
| (Wynne et al., 2004)21 | Lung abscess | Yes | NR | VOR | POS | NA | Favorable |
| (Wynne et al., 2004)21 | Lung abscess | Yes | NR | ITRA | POS | NA | Favorable |
| (Canales et al., 1998)22 | Pneumonia | Yes | NR | AMB | AMB+ITRA | NR | Favorable |
| (Songcharoen et al., 2011)23 | Pneumonia | NR | NR | NR | POS | NR | Favorable |
| ^24^(Almeida Júnior et al., 2014) | Pneumonia | Yes | NR | NR | VOR | NR | Favorable |
| ^24^(Almeida Júnior et al., 2014) | Mediastinitis | Yes | NR | ITRA | VOR | NR | Favorable |
| (Kahana et al., 2003)25 | Intra-abdominal infection | NR | NR | NR | AMB | NR | Favorable |
| (Biasoli et al., 2008)26 | Intra-abdominal infection | Yes | NR | NR | AMB, FLU+CAS | NR | Unfavorable |
| (Fadhil et al., 2011)27 | Intra-abdominal infection | NR | NR | NR | POS | NR | Favorable |
| (Anuradha et al., 2000)28 | Peritonitis | Yes | *Tenkoff* | NR | FLU | Yes | Favorable |
| (Chen et al., 2013)29 | Peritonitis | NR | *Tenkoff* | FLU | L-AMB^21^ | Yes | Favorable |
| ^30^(Macêdo et al., 2011) | Esophagitis | NR | NA | NR | FLU | NA | Not favorable |
| (Chakrabarti et al., 2002)31 | Generalized lymphadenopathy | NR | NR | NR | L-AMB+5-FC^22^ | NA | Not Favorable |
| (Basiri et al., 2012)32 | Brain abscess | No | No | No | AMB | NR | Favorable |
| (Ogura et al., 2012)33 | Granulomatous interstitial nephritis | Yes | NR | No | AMB | NR | Unfavorable |

^1^ CSF: cerebral spinal fluid; ^2^ NR: not reported; ^3^ ARF: acute renal failure; ^4^ BAL: bronchoalveolar lavage; ^5^ COPD: chronic obstructive pulmonary disease; ^6^ CONS: coagulase-negative *Staphylococcus* species; ^7^ anti-GXM: cryptococcal antigen detection assay [glucuronoxylomannan]; ^8^ GM: galactomannan detection assay; ^9^ CRF: chronic renal failure; ^10^ CM: collection from mediastinum; ^11^ GB: gallbladder; ^12^ PF: peritoneal fluid; ^13^ AB: previous antibiotic therapy; ^14^ BRT: breakthrough infection; ^15^ AMB: amphotericin B deoxicolate; ^16^CVC: central venous catheter; ^17^ FLU: fluconazole; ^18^ CAS: caspofungina; ^19^VOR: voriconazole; ^15^cryptococcal antigen detection assay (glucuronoxylomannan); ^16^galactomannan detection assay; ^20^ POS: posaconazole; ^21^ L-AMB: liposomal amphotericin B; ^22^ 5-FC: 5-flurocytosine.

**References**

Abdala, E., Lopes, R. I., Chaves, C. N., Heins-Vaccari, E. M., and Shikanai-Yasuda, M. A. (2005). Trichosporon asahii fatal infection in a non-neutropenic patient after orthotopic liver transplantation. *Transpl. Infect. Dis. Off. J. Transplant. Soc.* 7, 162–165. doi:10.1111/j.1399-3062.2005.00104.x.

Abliz, P., Fukushima, K., Takizawa, K., Yang, R., Li, R., and Nishimura, K. (2002). Identification of the first isolates of Trichosporon asahii var asahii from disseminated trichosporonosis in China. *Diagn. Microbiol. Infect. Dis.* 44, 17–22.

Almeida Júnior, J. N., Song, A. T. W., Campos, S. V., Strabelli, T. M. V., Del Negro, G. M., Figueiredo, D. S. Y., et al. (2014). Invasive Trichosporon infection in solid organ transplant patients: a report of two cases identified using IGS1 ribosomal DNA sequencing and a review of the literature. *Transpl. Infect. Dis. Off. J. Transplant. Soc.* doi:10.1111/tid.12179.

Anuradha, S., Chatterjee, A., Bajaj, J., Singh, N. P., Agarwal, S. K., and Kaur, R. (2000). Trichosporon beigelii peritonitis in a HIV-positive patient on continuous ambulatory peritoneal dialysis. *J. Assoc. Physicians India* 48, 1022–1024.

Basiri, K., Meidani, M., Rezaie, F., Soheilnader, S., and Fatehi, F. (2012). A rare case of Trichosporon brain abscess, successfully treated with surgical excision and antifungal agents. *Neurol. Neurochir. Pol.* 46, 92–95.

Biasoli, M. S., Carlson, D., Chiganer, G. J., Parodi, R., Greca, A., Tosello, M. E., et al. (2008). Systemic infection caused by Trichosporon asahii in a patient with liver transplant. *Med. Mycol. Off. Publ. Int. Soc. Hum. Anim. Mycol.* 46, 719–723. doi:10.1080/13693780802232928.

Canales, M. A., Sevilla, J., Ojeda Gutierrez, E., and Hernández Navarro, F. (1998). Successful treatment of Trichosporon beigelii pneumonia with itraconazole. *Clin. Infect. Dis. Off. Publ. Infect. Dis. Soc. Am.* 26, 999–1000.

Chagas-Neto, T. C., Chaves, G. M., Melo, A. S. A., and Colombo, A. L. (2009). Bloodstream infections due to Trichosporon spp.: species distribution, Trichosporon asahii genotypes determined on the basis of ribosomal DNA intergenic spacer 1 sequencing, and antifungal susceptibility testing. *J. Clin. Microbiol.* 47, 1074–1081. doi:10.1128/JCM.01614-08.

Chaitanya, V., Lakshmi, B. S., Kumar, A. C. V., Reddy, M. H. K., Ram, R., and Kumar, V. S. (2015). Disseminated Trichosporon infection in a renal transplant recipient. *Transpl. Infect. Dis. Off. J. Transplant. Soc.* 17, 605–609. doi:10.1111/tid.12412.

Chakrabarti, A., Marhawa, R. K., Mondal, R., Trehan, A., Gupta, S., Rao Raman, D. S. V., et al. (2002). Generalized lymphadenopathy caused by Trichosporon asahii in a patient with Job’s syndrome. *Med. Mycol.* 40, 83–86.

Chen, Y. T., Yang, W. C., Chen, T. W., and Lin, C. C. (2013). Trichosporon mucoides peritonitis in a continuous ambulatory peritoneal dialysis patient. *Perit. Dial. Int. J. Int. Soc. Perit. Dial.* 33, 341–342. doi:10.3747/pdi.2012.00146.

David, C., Martin, D. B., Deng, A., and Cooper, J. Z. (2008). Disseminated Trichosporon inkin and Histoplasma capsulatum in a patient with newly diagnosed AIDS. *J. Am. Acad. Dermatol.* 59, S13–15. doi:10.1016/j.jaad.2007.08.027.

Ebright, J. R., Fairfax, M. R., and Vazquez, J. A. (2001). Trichosporon asahii, a non-Candida yeast that caused fatal septic shock in a patient without cancer or neutropenia. *Clin. Infect. Dis. Off. Publ. Infect. Dis. Soc. Am.* 33, E28–30. doi:10.1086/322640.

Fadhil, R. A. S., Al-Thani, H., Al-Maslamani, Y., and Ali, O. (2011). Trichosporon fungal arteritis causing rupture of vascular anastamosis after commercial kidney transplantation: a case report and review of literature. *Transplant. Proc.* 43, 657–659. doi:10.1016/j.transproceed.2011.01.082.

Gross, J. W., and Kan, V. L. (2008). Trichosporon asahii infection in an advanced AIDS patient and literature review. *AIDS Lond. Engl.* 22, 793–795. doi:10.1097/QAD.0b013e3282f51ecc.

Gunn, S. R., Reveles, X. T., Hamlington, J. D., Sadkowski, L. C., Johnson-Pais, T. L., and Jorgensen, J. H. (2006). Use of DNA sequencing analysis to confirm fungemia due to Trichosporon dermatis in a pediatric patient. *J. Clin. Microbiol.* 44, 1175–1177. doi:10.1128/JCM.44.3.1175-1177.2006.

Hirschi, S., Letscher-Bru, V., Pottecher, J., Lannes, B., Jeung, M. Y., Degot, T., et al. (2012). Disseminated Trichosporon mycotoxinivorans, Aspergillus fumigatus, and Scedosporium apiospermum coinfection after lung and liver transplantation in a cystic fibrosis patient. *J. Clin. Microbiol.* 50, 4168–4170. doi:10.1128/JCM.01928-12.

Kahana, D. D., Cass, O., Jessurun, J., Schwarzenberg, S. J., Sharp, H., and Khan, K. (2003). Sclerosing cholangitis associated with trichosporon infection and natural killer cell deficiency in an 8-year-old girl: *J. Pediatr. Gastroenterol. Nutr.* 37, 620–623.

Karabay, O., Madariaga, M. G., Kocoglu, E., Ince, N., and Kandirali, E. (2006). Trichosporon asahii fungemia in a patient with non-hematological malignancy. *Jpn. J. Infect. Dis.* 59, 129–131.

Lacasse, A., and Cleveland, K. O. (2009). Trichosporon mucoides fungemia in a liver transplant recipient: case report and review. *Transpl. Infect. Dis. Off. J. Transplant. Soc.* 11, 155–159. doi:10.1111/j.1399-3062.2008.00355.x.

Lascaux, A. S., Bouscarat, F., Descamps, V., Casalino, E., Picard-Dahan, C., Crickx, B., et al. (1998). [Cutaneous manifestations during disseminated trichosporonosis in an AIDS patient]. *Ann. Dermatol. Vénéréologie* 125, 111–113.

Macêdo, D. P. C., de Oliveira, N. T., da Silva, V. K. A., de Almeida Farias, A. M., de Lima Neto, R. G., Wilheim, A. B., et al. (2011). Trichosporon inkin Esophagitis: An Uncommon Disease in a Patient with Pulmonary Cancer. *Mycopathologia* 171, 279–283. doi:10.1007/s11046-010-9367-5.

Nettles, R. E., Nichols, L. S., Bell-McGuinn, K., Pipeling, M. R., Scheel, P. J., Jr, and Merz, W. G. (2003). Successful treatment of Trichosporon mucoides infection with fluconazole in a heart and kidney transplant recipient. *Clin. Infect. Dis. Off. Publ. Infect. Dis. Soc. Am.* 36, E63–66. doi:10.1086/367665.

Nobrega de Almeida Júnior, J., Buccheri de Oliveira, R., Duarte, A., Lopes Motta, A., Rossi, F., Sachiko Yamamoto de Figueiredo, D., et al. (2015). Trichosporon inkin as an Emergent Pathogen in Patients With Severe Pemphigus. *JAMA Dermatol.* 151, 642–645. doi:10.1001/jamadermatol.2014.5462.

Ogura, M., Kagami, S., Nakao, M., Kono, M., Kanetsuna, Y., and Hosoya, T. (2012). Fungal granulomatous interstitial nephritis presenting as acute kidney injury diagnosed by renal histology including PCR assay. *Clin. Kidney J.* 5, 459–462. doi:10.1093/ckj/sfs103.

Ozkaya-Parlakay, A., Karadag-Oncel, E., Cengiz, A. B., Kara, A., Yigit, A., Gucer, S., et al. (2013). Trichosporon asahii sepsis in a patient with pediatric malignancy. *J. Microbiol. Immunol. Infect. Wei Mian Yu Gan Ran Za Zhi*. doi:10.1016/j.jmii.2013.01.003.

Piwoz, J. A., Stadtmauer, G. J., Bottone, E. J., Weitzman, I., Shlasko, E., and Cummingham-Rundles, C. (2000). Trichosporon inkin lung abscesses presenting as a penetrating chest wall mass. *Pediatr. Infect. Dis. J.* 19, 1025–1027.

Rodrigues, G. da S., de Faria, R. R. U., Guazzelli, L. S., Oliveira, F. de M., and Severo, L. C. (2006). [Nosocomial infection due to Trichosporon asahii: clinical revision of 22 cases]. *Rev. Iberoam. Micol.* 23, 85–89.

Servonnet, A., Bourgault, M., Trueba, F., Sarret, D., and Nicand, E. (2010). [Disseminated Trichosporon asahii infection]. *Ann. Biol. Clin. (Paris)* 68, 363–366. doi:10.1684/abc.2010.0444.

Songcharoen, S., Cleary, J. D., Jenkins, J., and DeShazo, M. (2011). T. asahii pulmonary infection as a complication of TNF-inhibitor and steroids: posaconazole pharmacotherapy and risk analysis. *J. Miss. State Med. Assoc.* 52, 339–343.

Tsai, M. S., Yang, Y. L., Wang, A. H., Wang, L. S., Lu, D. C. T., Liou, C. H., et al. (2012). Susceptibilities to amphotericin B, fluconazole and voriconazole of Trichosporon clinical isolates. *Mycopathologia* 174, 121–130. doi:10.1007/s11046-012-9525-z.

Wynne, S. M., Kwon-Chung, K. J., Shea, Y. R., Filie, A. C., Varma, A., Lupo, P., et al. (2004). Invasive infection with Trichosporon inkin in 2 siblings with chronic granulomatous disease. *J. Allergy Clin. Immunol.* 114, 1418–1424. doi:10.1016/j.jaci.2004.07.066.

Yang, M.-F., Gao, H.-N., and Li, L.-J. (2014). A fatal case of Trichosporon asahii fungemia and pneumonia in a kidney transplant recipient during caspofungin treatment. *Ther. Clin. Risk Manag.* 10, 759–762. doi:10.2147/TCRM.S67299.
