# Supplementary material for: Invasive Trichosporon Infection: a Systematic Review on a Re-emerging Fungal Pathogen
Source: Front Microbiol. 2016 Oct 17;7:1629. doi: 10.3389/fmicb.2016.01629 (PMC5065970; doi:10.3389/fmicb.2016.01629)
Supplement: Supplementary file 3 [file Table3.DOCX]

**Table S3**. Summary of the fifty-eight cases of invasive trichosporonosis in patients without immune disorders.

| Ref. | Type of Infection | Age/  Sex | Species | First  isolate | Other  sites | Serological tests | Other  organisms | Baseline  disease |
| --- | --- | --- | --- | --- | --- | --- | --- | --- |
| (Still et al., 1994) | Disseminated | 20/M | *Trichosporon* sp | Skin | Blood | NR^1^ | Bacillus  Staphylococcus  Proteus  Enterococcus | Burn, 31% of total body surface area |
| (Hajjeh and Blumberg, 1995) | Disseminated | 39/M | *Trichosporon* sp | Blood | NR | anti-GXM^2^ negative | NR | Burn, 45% of total body surface area |
| ^3^(Miró et al., 1994) | Disseminated | 20/M | *Trichosporon* sp | Blood | Urine,  bronchial  secretion | NR | NR | polytrauma |
| (Wang and Lin, 1999) | Disseminated | 76/M | *Trichosporon* sp | Blood | CVC^3^ tip | NR | NR | CRF^3^ |
| (Cawley et al., 2000) | Disseminated | 35/M | *Trichosporon* sp | blood | CVC tip, sputum, wounds | NR | VRE^6^, *Stenotrophomonas* | Burn, 85% of total body surface area |
| (Wolf et al., 2001)6 | Disseminated | 45/M | *T.asahii* | Urine | Skin biopsy | NR | *Candida parapsilosis* | Multiple penetrating trauma |
| (Kustimur et al., 2002) | Disseminated | 75/F | *T. asteroides* | Blood | Urine, tracheal aspirate, CVC tip | NR | NR | Pulmonary embolism |
| (Chitra et al., 2002) | Disseminated | 14/M | *Trichosporon* sp | Blood | NR | NR | NR | Cardiac surgery |
| ^9^(O’Gorman et al., 2006) | Disseminated | 71/M | *T. asahii* | Blood | NR | NR | NR | Polytrauma, abdominal surgery, ARF^7^ |
| (Kim et al., 2007) | Disseminated | 46/M | *T.asahii* | Blood | skin biopsy | NR | NR | No underlying disease |
| (Izumi et al., 2009) | Disseminated | 58/M | *T.asahii* | Blood | NR | GM^8^ and B-D-glucan positive | NR | Infective endocarditis |
| ^12^(Fagundes Júnior et al., 2008) | Disseminated | 85/F | *T. asahii* | Blood | Urine | NR | NR | Heart failure |
| (Chagas-Neto et al., 2009) | Disseminated | 39/M | *T.asahii* | Blood | NR | NR | NR | Heart failure |
| (Chagas-Neto et al., 2009) | Disseminated | 78/M | *T.asahii* | Blood | NR | NR | NR | Inflamatory gastrointestinal disease |
| (Chagas-Neto et al., 2009) | Disseminated | 3/M | *T.asahii* | Blood | NR | NR | NR | Burn |
| (Chagas-Neto et al., 2009) | Disseminated | 77/F | *T.asahii* | Blood | NR | NR | NR | Abdominal surgery |
| (Chagas-Neto et al., 2009) | Disseminated | 78/M | *T.asahii* | Blood | NR | NR | NR | Heart failure |
| (Chagas-Neto et al., 2009) | Disseminated | 2months/F | *T. asteroides* | Blood | NR | NR | NR | Galactosemia  congenital cytomegalovirus infection |
| (Chagas-Neto et al., 2009) | Disseminated | 54/F | *T. asteroides* | Blood | NR | NR | NR | Acute arterial embolism |
| (Chagas-Neto et al., 2009) | Disseminated | 43/M | *T. asteroides* | Blood | NR | NR | NR | Abdominal gunshot wound, splenectomy |
| (Chagas-Neto et al., 2009) | Disseminated | 53/F | *T. dermatis* | Blood | NR | NR | NR | Diabetes, hypertension |
| (Shang et al., 2010)14 | Disseminated | 53/F | *T.asahii* | Blood | No | NR | NR | CRF, secondary hemochromatosis |
| (Heslop et al., 2011) | Disseminated | 44/F | *T.asahii* | Skin | Brain | NR | NR | Burn, 50% of total body surface area |
| (Liao et al., 2012) | Disseminated | 43/F | *T.asahii* | Blood | NR | NR | NR | Acute virus myocarditis, ARF, corticosteroids |
| (Padhi et al., 2014) | Disseminated | 47/M | *T. mucoides/*  *dermatis* | Sputum, urine | Blood | NR | NR | Diabetes |
| (Negi et al., 2015) | Disseminated | 14/F | *T. asahii* | Blood | Urine | NR | NR | Diabetes, ARF |
| (De Saedeleer et al., 1994) | Peritonitis | 66/M | *Trichosporon* sp | PF^9^ | NR | NR | NR | CRF |
| (Lopes et al., 1994) | Peritonitis | 49/F | *Trichosporon* sp | PF | NR | NR | NR | CRF |
| (Lopes et al., 1995) | Peritonitis | 2/M | *Trichosporon* sp | PF | NR | NR | NR | Congenital malformations, CRF |
| (Melez et al., 1995) | Peritonitis | 18/M | *Trichosporon* sp | PF | NR | NR | No | CRF |
| (Kouppari et al., 1997) | Peritonitis | 4/M | *Trichosporon* sp | PF | NR | NR | NR | CRF |
| (Lopes et al., 1997) | Peritonitis | 45/M | *T. inkin* | PF | NR | NR | NR | CRF |
| (Wolf et al., 2001) | Peritonitis | 57/F | *T.asahii* | PF | NR | NR | NR | Diabetes, CRF |
| (Madariaga et al., 2003) | Peritonitis | 49/F | *T. inkin* | PF | Tenckhoff | NR | NR | CRF |
| (Crowther et al., 2003) | Peritonitis | 45/M | *T. inkin* | PF | NR | NR | NR | CRF |
| (Rodrigues et al., 2006) | Peritonitis | 6/M | *T.asahii* | PF | NR | NR | NR | CRF |
| (Rodrigues et al., 2006) | Peritonitis | 61/M | *T.asahii* | PF | NR | NR | NR | Cirrhosis, CRF, corticosteroids |
| (Jian et al., 2008) | Peritonitis | 49/F | *T.asahii* | PF | NR | NR | NR | CRF |
| (Sidarous et al., 1994) | Endocarditis | 77/M | *Trichosporon* sp | Blood | Spleen, aortic valve | NR | NR | Aortic valve replacement surgery |
| (Miralles et al., 1994) | Endocarditis | 44/M | *Trichosporon* sp | Embolic fragments | Aortic valve, blood culture | NR | NR | Aortic valve replacement surgery |
| (Chaumentin et al., 1996) | Endocarditis | 46/F | *T. inkin* | Mitral valve | Embolic material | NR | NR | Mitral valve replacement surgery |
| (Mooty et al., 2001) | Endocarditis | 62/F | *Trichosporon* sp | Mitral valve | NR | NR | NR | Mitral valve replacement surgery |
| (Ramos et al., 2004) | Endocarditis | 52/M | *T. inkin* | Blood | NR | NR | NR | Aortic valve replacement surgery |
| (Hickey et al., 2009) | Pneumonia | 20/M | *T. mycotoxinovorans* | Sputum | Tracheal aspirates, BAL^10^, lung tissue | NR | *Staphylococcus aureus* *Candida albicans* | Cystic fibrosis |
| (Tsai et al., 2011) | Pneumonia | 69/F | *T. asahii* | BAL | NR | NR | NR | Diabetes |
| (Lo Passo et al., 2001) | Esophagitis | 53/M | *T. asahii* | Esophagus biopsy | NR | NR | NR | No underlyng disease, heavy smoker |
| (Lo Passo et al., 2001) | Esophagitis | 32/F | *T. asahii* | Esophagus biopsy | Oesophageal brushing | NR | NR | No underlyng disease |
| (Kim et al., 2008) | Spondylodiscitis | 42/M | *T. asahii* | Vertebral abscess | NR | NR | NR | Lumbar disc herniation corretion |
| (Mathews and Prabhakar, 1995) | Meningitis | 36/F | *Trichosporon* sp | CSF^11^ | NR | NR | NR | Chronic back pain after trauma |
| (Kumar et al., 2015) | Meningitis | 18/M | *T. asahii* | CSF | NR | NR | NR | Chronic back pain |
| (Slocumb et al., 2010) | Endophthalmitis | 82/F | *T. asahii* | Vitreous biopsy | NR | NR | NR | Cataract surgery |
|  | Endophthalmitis | 58/M | *Trichosporon* sp | Vitreous biopsy | NR | NR | *Acremonium* sp | Cataract surgery |
| (Spirn et al., 2003) | Endophthalmitis | 72/M | *T. asahii* | Vitreous tap | NR | NR | NR | Cataract surgery |
| (Gonul et al., 2015) | Breast implant infection | 37/F | *Trichosporon* sp | Abscess around the implant | NR | NR | NR | Breast implant surgery |
| (Reddy et al., 2002) | Breast implant infection | 27/F | *Trichosporon* sp | Abscess around the implant | NR | NR | NR | Breast implant surgery |
| (Tian et al., 2007) | Knee implant infection | 73/F | *T. asahii* | Intra-articular fluid | NR | NR | NR | Knee implant surgery |

Table S3 continued.

| Ref. | Type of Infection | AB^12^ | Invasive disposal | BRT^13^ | Treatment | Invasive disposal  removal | Outcome |
| --- | --- | --- | --- | --- | --- | --- | --- |
| (Still et al., 1994) | Disseminated | Yes | NR | NR | AMB^14^ | NR | Favorable |
| (Hajjeh and Blumberg, 1995) | Disseminated | Yes | CVC | FLU^15^ | AMB+  5-FC^16^ | Yes | Favorable |
| (Miró et al., 1994) | Disseminated | Yes | CVC | NR | AMB | NR | Favorable |
| (Wang and Lin, 1999) | Disseminated | Yes | CVC | NR | AMB | Yes | Favorable |
| (Cawley et al., 2000) | Disseminated | Yes | CVC | FLU | AMB | Yes | Unfavorable |
| (Wolf et al., 2001) | Disseminated | Yes | CVC | NR | AMB | NR | Favorable |
| (Kustimur et al., 2002) | Disseminated | Yes | CVC | NR | AMB | Yes | Unfavorable |
| (Chitra et al., 2002) | Disseminated | NR | CVC | NR | FLU | NR | Unfavorable |
| (O’Gorman et al., 2006) | Disseminated | Yes | CVC | AMB+  MIC^8^ | AMB+  MIC | Yes | Unfavorable |
| (Kim et al., 2007) | Disseminated | NR | NR | NR | AMB | NR | Favorable |
| (Izumi et al., 2009) | Disseminated | Yes | NR | NR | FLU | NR | Favorable |
| (Fagundes Júnior et al., 2008) | Disseminated | Yes | NR | NR | L-AMB^17^ | NR | Unfavorable |
| (Chagas-Neto et al., 2009) | Disseminated | NR | CVC | NR | AMB | NR | Unfavorable |
| (Chagas-Neto et al., 2009) | Disseminated | NR | CVC | NR | AMB | NR | Unfavorable |
| (Chagas-Neto et al., 2009) | Disseminated | NR | CVC | NR | AMB | NR | Favorable |
| (Chagas-Neto et al., 2009) | Disseminated | NR | CVC | NR | No | NR | Unfavorable |
| (Chagas-Neto et al., 2009) | Disseminated | NR | CVC | NR | No | NR | Favorable |
| (Chagas-Neto et al., 2009) | Disseminated | Yes | CVC | NR | AMB | NR | Favorable |
| (Chagas-Neto et al., 2009) | Disseminated | NR | CVC | NR | FLU | NR | Favorable |
| (Chagas-Neto et al., 2009) | Disseminated | Yes | CVC | NR | AMB  +FLU | NR | Favorable |
| (Chagas-Neto et al., 2009) | Disseminated | Yes | CVC | NR | AMB  +FLU | NR | Unfavorable |
| (Shang et al., 2010) | Disseminated | Yes | CVC | NR | AMB  +VOR^18^ | NR | Favorable |
| (Heslop et al., 2011) | Disseminated | Yes | NR | NR | NR | NR | Unfavorable |
| (Liao et al., 2012) | Disseminated | Yes | CVC | CAS^19^ | VOR | Yes | Unfavorable |
| (Padhi et al., 2014) | Disseminated | NR | NR | NR | FLU | NR | Favorable |
| (Negi et al., 2015) | Disseminated | Yes | NR | NR | L-AMB | NR | Favorable |
| (De Saedeleer et al., 1994) | Peritonitis | Yes | Tenckhoff | NR | FLU | Yes | Favorable |
| (Lopes et al., 1994) | Peritonitis | NR | Tenckhoff | NR | FLU | Yes | Favorable |
| (Lopes et al., 1995) | Peritonitis | Yes | Tenckhoff | No | AMB | Yes | Favorable |
| (Melez et al., 1995) | Peritonitis | Yes | Tenckhoff | No | FLU | Yes | Favorable |
| (Kouppari et al., 1997) | Peritonitis | Yes | Tenckhoff | FLU+  5-FC | AMB | Yes | Favorable |
| (Lopes et al., 1997) | Peritonitis | Yes | Tenckhoff | No | FLU | Yes | Favorable |
| (Wolf et al., 2001) | Peritonitis | Yes | Tenckhoff | No | AMB | Yes | Favorable |
| (Madariaga et al., 2003) | Peritonitis | Yes | Tenckhoff | No | CAS | Yes | Favorable |
| (Crowther et al., 2003) | Peritonitis | Yes | Tenckhoff | No | FLU  +5-FC | Yes | Favorable |
| (Rodrigues et al., 2006) | Peritonitis | Yes | Tenckhoff | No | AMB | NR | Favorable |
| (Rodrigues et al., 2006) | Peritonitis | Yes | Tenckhoff | NR | AMB | NR | Unfavorable |
| (Jian et al., 2008) | Peritonitis | Yes | Tenckhoff | NR | FLU | Yes | Favorable |
| (Sidarous et al., 1994) | Endocarditis | Yes | NR | NR | AMB | NR | Favorable |
| (Miralles et al., 1994) | Endocarditis | Yes | NR | AMB | FLU | NR | Unfavorable |
| (Chaumentin et al., 1996) | Endocarditis | NR | NR | NR | AMB+  ITRA^20^ | NR | Favorable |
| (Mooty et al., 2001) | Endocarditis | NR | NR | NR | No | NR | Unfavorable |
| (Ramos et al., 2004) | Endocarditis | Yes | NR | NR | No | NR | Unfavorable |
| (Hickey et al., 2009) | Pneumonia | Yes | NR | NR | VOR | NR | Unfavorable |
| (Tsai et al., 2011) | Pneumonia | Yes | NR | NR | FLU | NR | Favorable |
| (Lo Passo et al., 2001) | Esophagitis | Yes | NA | NR | FLU | NR | Favorable |
| (Lo Passo et al., 2001) | Esophagitis | Yes | NA | NR | ITRA | NR | Favorable |
| (Kim et al., 2008) | Spondylodiscitis | Yes | NA | NR | FLU | NR | Favorable |
| (Mathews and Prabhakar, 1995) | Meningitis | Yes | NR | NR | No | NR | Unfavorable |
| (Kumar et al., 2015) | Meningitis | Yes | NR | NR | L-AMB | NR | Unfavorable |
| (Slocumb et al., 2010) | Endophthalmitis | NR | NR | NR | VOR | NR | Favorable |
|  |  |  |  |  |  |  |  |
| (Spirn et al., 2003) | Endophthalmitis | NR | NR | AMB | FLU | NR | Favorable |
| (Gonul et al., 2015) | Endophthalmitis | Yes | NR | NR | VOR | NR | Favorable |
| (Reddy et al., 2002) | Breast implant infection | NR | NR | NR | FLU | NR | Favorable |
| (Tian et al., 2007) | Breast implant infection | NR | NR | NR | FLU | NR | Favorable |
| (Zuo et al., 2015) | Knee implant infection | Yes | NR | NR | AMB, VOR | NR | Favorable |

^1^ NR: not reported; ^2^ GXM: cryptococcal antigen detection assay [glucuronoxylomannan]; ^3^ CVC: central venous catheter; ^4^ CRF: chronic renal failure; ^5^ VRE: vancomycin-resistant *Enterococcus*; ^6^ ARF: acute renal failure; ^8^ GM: galactomannan detection assay; ^9^peritoneal fluid; ^10^bronchoalveolar lavage; ^11^cerebral spinal fluid; ^12^ BI: previous antibiotic therapy; ^13^BRT: breakthrough infection; ^14^AMB: amphotericin B deoxicolate; ^15^FLU: fluconazole; ^16^5-FC: 5-fluorocytosine; ^17^MIC: micafungin; ^18^L-AMB: liposomal amphotericin B; ^19^VOR: voriconazole; ^20^CAS: caspofungine; ^21^ITRA: itraconazole.

**References**

Cawley, M. J., Braxton, G. R., Haith, L. R., Reilly, K. J., Guilday, R. E., and Patton, M. L. (2000). Trichosporon beigelii infection: experience in a regional burn center. *Burns J. Int. Soc. Burn Inj.* 26, 483–486.

Chagas-Neto, T. C., Chaves, G. M., Melo, A. S. A., and Colombo, A. L. (2009). Bloodstream infections due to Trichosporon spp.: species distribution, Trichosporon asahii genotypes determined on the basis of ribosomal DNA intergenic spacer 1 sequencing, and antifungal susceptibility testing. *J. Clin. Microbiol.* 47, 1074–1081. doi:10.1128/JCM.01614-08.

Chaumentin, G., Boibieux, A., Piens, M. A., Douchet, C., Buttard, P., Bertrand, J. L., et al. (1996). Trichosporon inkin endocarditis: short-term evolution and clinical report. *Clin. Infect. Dis. Off. Publ. Infect. Dis. Soc. Am.* 23, 396–397.

Chitra, A. K., Verghese, S., Fernandez, M., Mohan, A., Abraham, A., and Methew, T. (2002). Trichosporonosis due to Trichosporon beigelli in two hospitalized patients. *Indian J. Pathol. Microbiol.* 45, 337–339.

Crowther, K. S., Webb, A. T., and McWhinney, P. H. (2003). Trichosporon inkin peritonitis in a patient on continuous ambulatory peritoneal dialysis returning from the Caribbean. *Clin. Nephrol.* 59, 69–70.

Fagundes Júnior, A. A. de P., Carvalho, R. T. de, Focaccia, R., Fernandez, J. G., Araújo, H. B. N. de, Strabelli, T. M. V., et al. (2008). [Trichosporon asahii an emerging etiologic agent of fungal infection and colonization in heart failure patients in intensive care unit: case report and literature review]. *Rev. Bras. Ter. Intensiva* 20, 106–109.

Gonul, S., Gedik, S., Ozturk, B. T., Bakbak, B., Koktekir, B. E., Okudan, S., et al. (2015). Postoperative fungal endophthalmitis caused by Trichosporon asahii treated with voriconazole. *Arq. Bras. Oftalmol.* 78, 252–254. doi:10.5935/0004-2749.20150065.

Hajjeh, R. A., and Blumberg, H. M. (1995). Bloodstream infection due to Trichosporon beigelii in a burn patient: case report and review of therapy. *Clin. Infect. Dis. Off. Publ. Infect. Dis. Soc. Am.* 20, 913–916.

Heslop, O. D., Nyi Nyi, M.-P., Abbott, S. P., Rainford, L. E., Castle, D. M., and Coard, K. C. M. (2011). Disseminated trichosporonosis in a burn patient: meningitis and cerebral abscess due to Trichosporon asahii. *J. Clin. Microbiol.* 49, 4405–4408. doi:10.1128/JCM.05028-11.

Hickey, P. W., Sutton, D. A., Fothergill, A. W., Rinaldi, M. G., Wickes, B. L., Schmidt, H. J., et al. (2009). Trichosporon mycotoxinivorans, a novel respiratory pathogen in patients with cystic fibrosis. *J. Clin. Microbiol.* 47, 3091–3097. doi:10.1128/JCM.00460-09.

Izumi, K., Hisata, Y., and Hazama, S. (2009). A rare case of infective endocarditis complicated by Trichosporon asahii fungemia treated by surgery. *Ann. Thorac. Cardiovasc. Surg. Off. J. Assoc. Thorac. Cardiovasc. Surg. Asia* 15, 350–353.

Jian, D. Y., Yang, W. C., Chen, T. W., and Lin, C. C. (2008). Trichosporon asahii following polymicrobial infection in peritoneal dialysis-associated peritonitis. *Perit. Dial. Int. J. Int. Soc. Perit. Dial.* 28, 100–101.

Kim, K.-W., Ha, K.-Y., Kim, M.-S., Choi, S.-M., and Lee, J.-S. (2008). Postoperative Trichosporon asahii spondylodiscitis after open lumbar discectomy: a case report. *Spine* 33, E116–120. doi:10.1097/BRS.0b013e3181642a7c.

Kim, Y. J., Kim, S. I., Kim, Y. R., Park, Y. M., Park, Y. J., and Kang, M. W. (2007). Successful treatment of septic shock with purpura fulminans caused by Trichosporon asahii in an immunocompetent patient. *Ann. Clin. Lab. Sci.* 37, 366–369.

Kouppari, G., Stephanidis, K., Zaphiropoulou, A., Siapera, D., and Deliyianni, V. (1997). Trichosporon beigelii peritonitis in a child on continuous ambulatory peritoneal dialysis. *Clin. Microbiol. Infect. Off. Publ. Eur. Soc. Clin. Microbiol. Infect. Dis.* 3, 509–510.

Kumar, A., Udayakumaran, S., Babu, R., Rajamma, B. M., Prakash, A., Panikar, D., et al. (2015). Trichosporon asahii infection presenting as chronic meningo-ventriculitis and intra ventricular fungal ball: a case report and literature review. *Mycoses* 58, 99–103. doi:10.1111/myc.12282.

Kustimur, S., Kalkanci, A., Caglar, K., Dizbay, M., Aktas, F., and Sugita, T. (2002). Nosocomial fungemia due to Trichosporon asteroides: firstly described bloodstream infection. *Diagn. Microbiol. Infect. Dis.* 43, 167–170.

Liao, Y., Hartmann, T., Zheng, T., Yang, R.-Y., Ao, J.-H., and Wang, W.-L. (2012). Breakthrough trichosporonosis in patients receiving echinocandins: case report and literature review. *Chin. Med. J. (Engl.)* 125, 2632–2635.

Lopes, J. O., Alves, S. H., Benevenga, J. P., Rosa, A. C., and Gomez, V. C. (1994). Trichosporon beigelii peritonitis associated with continuous ambulatory peritoneal dialysis. *Rev. Inst. Med. Trop. São Paulo* 36, 121–123.

Lopes, J. O., Alves, S. H., Klock, C., Oliveira, L. T., and Dal Forno, N. R. (1997). Trichosporon inkin peritonitis during continuous ambulatory peritoneal dialysis with bibliography review. *Mycopathologia* 139, 15–18.

Lopes, J. O., Silva, C. B., Kmohan, C., Salla, A., and Righi, R. A. (1995). [Trichosporon beigelii peritonitis in a child during treatment by continuous ambulatory peritoneal dialysis]. *J. Pediatr. (Rio J.)* 71, 341–343.

Madariaga, M. G., Tenorio, A., and Proia, L. (2003). Trichosporon inkin peritonitis treated with caspofungin. *J. Clin. Microbiol.* 41, 5827–5829.

Mathews, M. S., and Prabhakar, S. (1995). Chronic meningitis caused by Trichosporon beigelii in India. *Mycoses* 38, 125–126.

Melez, K. A., Cherry, J., Sanchez, C., Ettinger, R. B., and Walsh, T. J. (1995). Successful outpatient treatment of Trichosporon beigelii peritonitis with oral fluconazole. *Pediatr. Infect. Dis. J.* 14, 1110–1113.

Miralles, A., Quiroga, J., Farinola, T., Obi, C., Saura, E., Fontanillas, C., et al. (1994). Recurrent Trichosporon beigelii endocarditis after aortic valve replacement. *Cardiovasc. Surg. Lond. Engl.* 2, 119–123.

Miró, O., Sacanella, E., Nadal, P., Lluch, M. M., Nicolás, J. M., Millá, J., et al. (1994). Trichosporon beigelii fungemia and metastatic pneumonia in a trauma patient. *Eur. J. Clin. Microbiol. Infect. Dis. Off. Publ. Eur. Soc. Clin. Microbiol.* 13, 604–606.

Mooty, M. Y., Kanj, S. S., Obeid, M. Y., Hassan, G. Y., and Araj, G. F. (2001). A case of Trichosporon beigelii endocarditis. *Eur. J. Clin. Microbiol. Infect. Dis. Off. Publ. Eur. Soc. Clin. Microbiol.* 20, 139–142.

Negi, V., Sharma, M., Juyal, D., Kotian, S., and Sharma, N. (2015). Disseminated trichosporonosis due to Trichosporon asahii in a diabetic patient. *Indian J. Pathol. Microbiol.* 58, 246–248. doi:10.4103/0377-4929.155333.

O’Gorman, C., McMullan, R., Webb, C., and Bedi, A. (2006). Trichosporon Asahii. Blood-stream Infection in a non-cancer patient receiving Combination Antifungal Therapy. *Ulster Med. J.* 75, 226–227.

Padhi, S., Dash, M., Pattanaik, S., and Sahu, S. (2014). Fungemia due to Trichosporon mucoides in a diabetes mellitus patient: a rare case report. *Indian J. Med. Microbiol.* 32, 72–74. doi:10.4103/0255-0857.124324.

Lo Passo, C., Pernice, I., Celeste, A., Perdichizzi, G., and Todaro-Luck, F. (2001). Transmission of Trichosporon asahii oesophagitis by a contaminated endoscope. *Mycoses* 44, 13–21.

Ramos, J. M., Cuenca-Estrella, M., Gutierrez, F., Elia, M., and Rodriguez-Tudela, J. L. (2004). Clinical case of endocarditis due to Trichosporon inkin and antifungal susceptibility profile of the organism. *J. Clin. Microbiol.* 42, 2341–2344.

Reddy, B. T., Torres, H. A., and Kontoyiannis, D. P. (2002). Breast implant infection caused by Trichosporon beigelii. *Scand. J. Infect. Dis.* 34, 143–144. doi:10.1080/00365540110026895.

Rodrigues, G. da S., de Faria, R. R. U., Guazzelli, L. S., Oliveira, F. de M., and Severo, L. C. (2006). [Nosocomial infection due to Trichosporon asahii: clinical revision of 22 cases]. *Rev. Iberoam. Micol.* 23, 85–89.

De Saedeleer, B., Sennesael, J., Van der Niepen, P., and Verbeelen, D. (1994). Intraperitoneal fluconazole therapy for Trichosporon cutaneum peritonitis in continuous ambulatory peritoneal dialysis. *Nephrol. Dial. Transplant. Off. Publ. Eur. Dial. Transpl. Assoc. - Eur. Ren. Assoc.* 9, 1658–1659.

Shang, S.-T., Yang, Y.-S., and Peng, M.-Y. (2010). Nosocomial Trichosporon asahii fungemia in a patient with secondary hemochromatosis: a rare case report. *J. Microbiol. Immunol. Infect. Wei Mian Yu Gan Ran Za Zhi* 43, 77–80. doi:10.1016/S1684-1182(10)60012-6.

Sidarous, M. G., O’Reilly, M. V., and Cherubin, C. E. (1994). A case of Trichosporon beigelii endocarditis 8 years after aortic valve replacement. *Clin. Cardiol.* 17, 215–219.

Slocumb, R. W., Elner, S. G., and Hall, E. F. (2010). Chronic postoperative fungal endophthalmitis caused by trichosporon asahii. *Retin. Cases Brief Rep.* 4, 366–367. doi:10.1097/ICB.0b013e3181b5ef61.

Spirn, M. J., Roth, D. B., Yarian, D. L., and Green, S. N. (2003). Postoperative fungal endophthalmitis caused by Trichosporon beigelii resistant to amphotericin B. *Retina Phila. Pa* 23, 404–405.

Still, J. M., Orlet, K., and Law, E. J. (1994). Trichosporon beigelii septicaemia in a burn patient. *Burns J. Int. Soc. Burn Inj.* 20, 467–468.

Tian, H. H., Tan, S. M., and Tay, K. H. (2007). Delayed fungal infection following augmentation mammoplasty in an immunocompetent host. *Singapore Med. J.* 48, 256–258.

Tsai, M.-J., Chang, W.-A., Tsai, K.-B., Chen, H.-C., Hwang, J.-J., and Huang, M.-S. (2011). Probable invasive pulmonary trichosporonosis in a diabetic patient. *Am. J. Respir. Crit. Care Med.* 184, 982. doi:10.1164/rccm.201101-0144IM.

Wang, H. Y., and Lin, J. L. (1999). Trichosporon beigelii fungaemia in a patient with haemodialysis. *Nephrol. Dial. Transplant. Off. Publ. Eur. Dial. Transpl. Assoc. - Eur. Ren. Assoc.* 14, 2017–2018.

Wolf, D. G., Falk, R., Hacham, M., Theelen, B., Boekhout, T., Scorzetti, G., et al. (2001). Multidrug-resistant Trichosporon asahii infection of nongranulocytopenic patients in three intensive care units. *J. Clin. Microbiol.* 39, 4420–4425. doi:10.1128/JCM.39.12.4420-4425.2001.

Zuo, Q., Dong, L., Mu, W., Zhou, L., Hu, T., and Zhang, H. (2015). Trichosporon asahii infection after total knee arthroplasty: A case report and review of the literature. *Can. J. Infect. Dis. Med. Microbiol. J. Can. Mal. Infect. Microbiol. Médicale AMMI Can.* 26, 47–51.
