# Supplementary material for: Invasive Trichosporon Infection: a Systematic Review on a Re-emerging Fungal Pathogen
Source: Front Microbiol. 2016 Oct 17;7:1629. doi: 10.3389/fmicb.2016.01629 (PMC5065970; doi:10.3389/fmicb.2016.01629)
Supplement: Supplementary file 4 [file Table4.DOCX]

**Table S4**. Summary of the twety-two cases of invasive trichosporonosis in neonates.

| Ref. | Weight/  Sex | Days of life | Species | First isolate | Other sites | Other  pathogens | Clinical conditions |
| --- | --- | --- | --- | --- | --- | --- | --- |
| (Yoss et al., 1997) | 530g/M | 10 | *Trichosporon sp.* | Blood | CVC^1^-tip, tracheal aspirate | NR^2^ | Preterm, PNA^3^, RDS^4^ |
| (Sweet and Reid, 1998) | 950g/F | 16 | *Trichosporon sp* | Blood | Urine,skin,peritoneal fluid,  endotracheal aspirate | No | Preterm, RDS, necrotising enterocolitis |
| (Salazar and Campbell, 2002) | 720g/M | 17 | *Trichosporon sp* | Blood | Urine  tracheal aspirate, peritoneal fluid | CONS^5^ | Preterm, PNA RDS, enterectomy |
| (Salazar and Campbell, 2002) | 960g/M | 15 | *Trichosporon sp.* | Blood | NR | NR | Preterm, PNA,RDS, enterectomy |
| (Panagopoulou et al., 2002) | 890/F | 11 | *T. asahii* | Blood | CVC-tip, perineal and pharingeal swab | Klebsiella pneumoniae | Preterm, PNA, RDS |
| (Gökahmetoğlu et al., 2002) | 1300g/F | 28 | *T. mucoides/*  *dermatis* | Blood | NR | No | Preterm |
| (Gökahmetoğlu et al., 2002) | 2300g/M | 3 | *T. mucoides/*  *dermatis* | Blood | NR | No | Preterm |
| (Gökahmetoğlu et al., 2002) | 1400g/M | 18 | *T. mucoides/*  *dermatis* | Blood | NR | No | Preterm |
| (Yildiran et al., 2003) | 1050g/F | 21 | *T. asahii* | Urine | Blood | No | Preterm |
| (Maheshwari et al., 2004) | 737g/F | 26 | *T. asahii* | Blood | Blood from CVC | CONS | Preterm, RDS, intraventricular hemorrhage |
| (Pereira et al., 2009) | 815g/M | 16 | *Trichosporon sp* | Blood | NR | NR | Preterm, PNA, RDS, enterectomy |
| (Chagas-Neto et al., 2009) | NR/F | 16 | *T. asahii* | Blood | NR | NR | Preterm |
| (Chagas-Neto et al., 2009) | NR/M | 84 | *T. asahii* | Blood | NR | NR | Preterm, enterectomy |
| (Chagas-Neto et al., 2009) | NR/F | 15 | *T. asteroides* | Blood | NR | NR | Preterm |
| (Vashishtha et al., 2012) | 2400g/NR | NR | *T. asahii* | Blood | NR | NR | SGA^6^, PNA |
| (Vashishtha et al., 2012) | 1200g/NR | NR | *T. asahii* | Blood | NR | NR | Preterm, SGA |
| (Vashishtha et al., 2012) | 1250g/NR | NR | *T. asahii* | Blood | NR | NR | Preterm, SGA, PNA, RDS |
| (Vashishtha et al., 2012) | 1080g/NR | 11 | *T. asahii* | Blood | NR | NR | Preterm, PNA |
| (Vashishtha et al., 2012) | 1720g/NR | 11 | *T. asahii* | Blood | NR | NR | Preterm, SGA |
| (Vashishtha et al., 2012) | 1235g/NR | NR | *T. asahii* | Blood | NR | NR | Preterm, PNA |
| (Vashishtha et al., 2012) | 1550g/NR | 7 | *T. asahii* | Blood | NR | NR | Preterm, SGA, PNA |
| (Vashishtha et al., 2012) | 2890g/NR | 5 | *T. asahii* | Blood | NR | NR | MSAF^7^, PNA |
| (Basu et al., 2015) | 920/M | 9 | *T. asahii* | Blood | NR | NR | Preterm, RDS |
| (Basu et al., 2015) | 980/M | 8 | *T. asahii* | Blood | NR | NR | Preterm, RDS |
| (Basu et al., 2015) | 900/F | 11 | *T. asahii* | Blood | NR | *Klebsiella pneumoniae* | Preterm, RDS |

Table S4 continued.

| Ref. | Previous antimicrobial therapy | Central venous catheter | Breakthrough infection | Treatment | CVC removal | Outcome |
| --- | --- | --- | --- | --- | --- | --- |
| (Yoss et al., 1997) | Yes | Yes | No | AMB^8^ | Yes | Unfavorable |
| (Sweet and Reid, 1998) | Yes | NR | No | L-AMB^9^ | NR | Favorable |
| (Salazar and Campbell, 2002) | Yes | Yes | No | AMB | NR | Favorable |
| (Salazar and Campbell, 2002) | Yes | NR | No | AMB | NR | Unfavorable |
| (Panagopoulou et al., 2002) | Yes | Yes | No | AMB | Yes | Favorable |
| (Gökahmetoğlu et al., 2002) | Yes | NR | No | FLU^10^ | NR | Favorable |
| (Gökahmetoğlu et al., 2002) | NR | NR | No | AMB | NR | Favorable |
| (Gökahmetoğlu et al., 2002) | Yes | NR | No | AMB | NR | Favorable |
| (Yildiran et al., 2003) | Yes | Yes | No | AMB | NR | Favorable |
| (Maheshwari et al., 2004) | Yes | Yes | AMB | AMB+5-FC^11^ | Yes | Favorable |
| (Pereira et al., 2009) | Yes | Yes | AMB | AMB+FLU | Yes | Unfavorable |
| (Chagas-Neto et al., 2009) | Yes | Yes | NR | AMB | NR | Unfavorable |
| (Chagas-Neto et al., 2009) | Yes | Yes | NR | AMB+FLU | NR | Favorable |
| (Chagas-Neto et al., 2009) | Yes | Yes | NR | AMB | NR | Favorable |
| (Vashishtha et al., 2012) | Yes | NR | NR | AMB | NR | Favorable |
| (Vashishtha et al., 2012) | Yes | NR | NR | AMB | NR | Unfavorable |
| (Vashishtha et al., 2012) | Yes | NR | NR | AMB | NR | Unfavorable |
| (Vashishtha et al., 2012) | Yes | NR | NR | L-AMB | NR | Unfavorable |
| (Vashishtha et al., 2012) | Yes | NR | NR | AMB | NR | Favorable |
| (Vashishtha et al., 2012) | Yes | NR | NR | AMB | NR | Unfavorable |
| (Vashishtha et al., 2012) | Yes | NR | NR | AMB | NR | Unfavorable |
| (Vashishtha et al., 2012) | Yes | NR | NR | AMB | NR | Unfavorable |
| (Basu et al., 2015) | Yes | Yes | FLU | L-AMB | NR | Unfavorable |
| (Basu et al., 2015) | Yes | Yes | NR | L-AMB | NR | Unfavorable |
| (Basu et al., 2015) | Yes | Yes | FLU | L-AMB | NR | Unfavorable |

^1^CVC: Central vascular catheter; ^2^NR: not reported; ^3^PNA: perinatal asphyxia; ^4^ RDS: respiratory distress syndrome;  ^5^CONS: coagulase negative *Staphylococcus*; ^6^SGA: small for gestational age; ^7^ MSAF: meconium stained amniotic fluid;  ^8^ AMB: amphotericin B deoxicolate;; ^9^ L-AMB: liposomal amphotericin B; ^10^ FLU: fluconazole; ^11^ 5-FC: 5-fluorocytosine.

**References**

Basu, S., Tilak, R., and Kumar, A. (2015). Multidrug-resistant Trichosporon: an unusual fungal sepsis in preterm neonates. *Pathog. Glob. Health* 109, 202–206. doi:10.1179/2047773215Y.0000000019.

Chagas-Neto, T. C., Chaves, G. M., Melo, A. S. A., and Colombo, A. L. (2009). Bloodstream infections due to Trichosporon spp.: species distribution, Trichosporon asahii genotypes determined on the basis of ribosomal DNA intergenic spacer 1 sequencing, and antifungal susceptibility testing. *J. Clin. Microbiol.* 47, 1074–1081. doi:10.1128/JCM.01614-08.

Gökahmetoğlu, S., Nedret Koç, A., Güneş, T., and Cetin, N. (2002). Case reports. Trichosporon mucoides infection in three premature newborns. *Mycoses* 45, 123–125.

Maheshwari, A., Stromquist, C. I., Pereda, L., and Emmanuel, P. J. (2004). Mixed infection with unusual fungi and staphylococcal species in two extremely premature neonates. *J. Perinatol. Off. J. Calif. Perinat. Assoc.* 24, 324–326. doi:10.1038/sj.jp.7211077.

Panagopoulou, P., Evdoridou, J., Bibashi, E., Filioti, J., Sofianou, D., Kremenopoulos, G., et al. (2002). Trichosporon asahii: an unusual cause of invasive infection in neonates. *Pediatr. Infect. Dis. J.* 21, 169–170.

Pereira, D. N., Nader, S. S., Nader, P., Martins, P. G., Furlan, S. P., and Hentges, C. R. (2009). Disseminated Trichosporon spp infection in preterm newborns: a case report. *J. Pediatr. (Rio J.)* 85, 459–461. doi:10.2223/JPED.1923.

Salazar, G. E., and Campbell, J. R. (2002). Trichosporonosis, an unusual fungal infection in neonates. *Pediatr. Infect. Dis. J.* 21, 161–165.

Sweet, D., and Reid, M. (1998). Disseminated neonatal Trichosporon beigelii infection: successful treatment with liposomal amphotericin B. *J. Infect.* 36, 120–121.

Vashishtha, V. M., Mittal, A., and Garg, A. (2012). A fatal outbreak of Trichosporon asahii sepsis in a neonatal intensive care Unit. *Indian Pediatr.* 49, 745–747.

Yildiran, A., Kücüködük, S., Saniç, A., Belet, N., and Güvenli, A. (2003). Disseminated Trichosporon asahii infection in a preterm. *Am. J. Perinatol.* 20, 269–271. doi:10.1055/s-2003-42337.

Yoss, B. S., Sautter, R. L., and Brenker, H. J. (1997). Trichosporon beigelii, a new neonatal pathogen. *Am. J. Perinatol.* 14, 113–117. doi:10.1055/s-2007-994109.
